# Molecular diagnostics for cutaneous leishmaniasis: progress towards fulfilling the WHO target product profile

**DOI:** 10.1017/S0031182025101467

**Published:** 2026-03

**Authors:** Jan Jarzabek, Paul William Denny

**Affiliations:** 1 Department of Biosciences, Durham University, Durham, UK; 2 Department of Chemistry, Durham University, Durham, UK

**Keywords:** cutaneous leishmaniasis, diagnosis, isothermal, point-of-care testing

## Abstract

Recently, the WHO published a Target Product Profile for a diagnostic test for cutaneous leishmaniasis (CL) and a Roadmap to 2030 for Neglected Tropical Diseases. The documents highlight that existing diagnostic tools for CL are insufficient, whilst setting clear goals for improved sensitivity and reduced cost. The need for species typing in diagnostics is also becoming more pressing with the emergence of drug-resistance, especially of *Leishmania tropica*. Serological tests are unable to do this, while techniques that can, like PCR, require complex and expensive machinery. Isothermal assays like LAMP offer a promising solution, but more work also remains, as few species-specific LAMP assays have been developed thus far and CL in Ethiopia is particularly neglected. Additionally, since the COVID-19 pandemic, many cheap isothermal diagnostic devices have been produced, which have yet to be tested in the diagnosis of CL. Finally, artificial intelligence presents another avenue for rapid diagnosis by image analysis. In this comprehensive review, we examine the opportunities and challenges inherent to diagnostic development for CL, a priority undertaking that still faces many developmental hurdles.

## Graphical abstract

**Figure:**
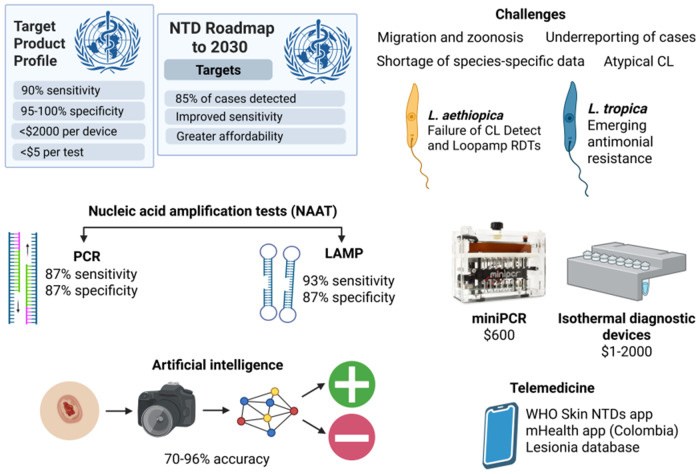

## Introduction

Leishmaniasis is one of 25 neglected tropical diseases (NTDs) characterised by their impact on 100’s of millions of the global poor and a chronic lack of attention and investment (World Health Organisation, 2021). Caused by the protist *Leishmania* spp., the disease has three primary clinical presentations: cutaneous (CL), mucocutaneous (MCL) and visceral (VL) (Georgiadou et al. 2015). CL involves lesions on the skin and accounts for the majority of cases (Aronson et al. 2016). MCL features infection of the mucous membranes of the respiratory tract, while VL is an infection of the internal organs such as the liver and spleen (Aronson et al. 2016). Currently, 49% countries reporting to the WHO are considered endemic for leishmaniasis, 45% for CL specifically (Jain et al. 2024b).

**Figure 1.**
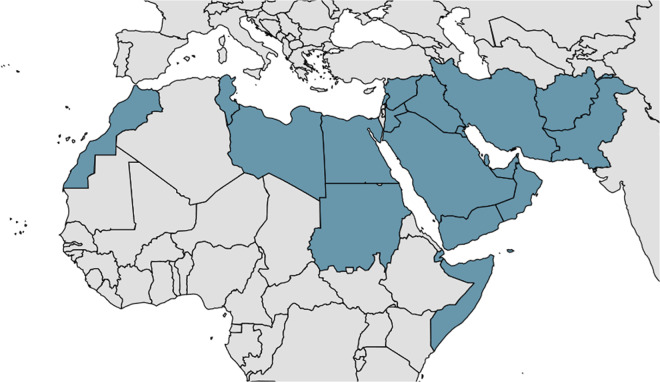
The Eastern Mediterranean Region (EMR), as defined by the WHO, comprising Afghanistan, Bahrain, Djibouti, Egypt, Iran, Iraq, Jordan, Kuwait, Lebanon, Libya, Morocco, Oman, Pakistan, Palestine, Qatar, Saudi Arabia, Somalia, Sudan, Syria, Tunisia, UAE, and Yemen. Figure 1 long description.

The most recent WHO figures (2023) report 272 000 cases of CL, with the vast majority (81%) occurring in the Eastern Mediterranean Region (Figure 1) (Jain et al. 2024b). This is a slight reduction from the peak of 281 000 cases in 2019; however, there has been an upward trend since 2014 (Jain et al. 2024b). At the same time, VL case numbers fell from 31 000 to 12 000 (Jain et al. 2024b). Another study reported a 95% reduction in VL incidence globally from 1990 to 2021 (Zhang et al. 2025c). This positive trend is due to public health interventions focused on VL, most notably the joint eradication programme in India, Bangladesh and Nepal (Thakur et al. 2020; Jain et al. 2024b). In fact, VL is one of eight NTDs which have been eliminated in at least one country, in this case Bangladesh (Hietanen et al. 2025). Unfortunately, CL remains neglected – a recent review found that the total burden of CL across North Africa and the Middle East had not changed from 1990 to 2021 (EL Banna et al. 2025). It has also been suggested that, due to the shortage of diagnostic tools, CL case numbers are underreported and could be as high as one million per annum (de Vries and Schallig, 2022).

The WHO NTD Road Map to 2030 identifies diagnostics as an area in CL research where critical action is required, with a need to improve the sensitivity and affordability and a goal of 85% of cases being detected in every endemic country by 2030 (World Health Organisation, 2021). A recent report concluded that these goals had not yet been met (Donadeu et al. 2025). Indeed, based on the latest figures from the WHO, only 27% of cases are being reported (Jain et al. 2024b). Challenges in CL diagnosis which may account for this failure include the relative lack of resources and medical infrastructure, political instability and the presence of multiple endemic species (Akhoundi et al. 2017; de Vries and Schallig, 2022). Additionally, CL lesions can be misdiagnosed as a number of communicable and non-communicable diseases such as cutaneous tuberculosis, psoriasis, purulent dermatitis and pyoderma gangrenosum due to similarities in appearance (Di Altobrando et al. 2021; Sikorska et al. 2022; Karaja et al. 2024; Shrestha et al. 2024). To address these challenges, a target product profile (TPP) by the Foundation for Innovative Diagnostics, in collaboration with the Drugs for Neglected Diseases initiative and the WHO outlines the desired characteristics for a CL diagnostic test (Cruz et al. 2019). The TPP lists criteria such as specificity (95–100%), sensitivity ( > 90%) and ease of use, both with respect to performing the test and in sample collection and preparation. The TPP also lists the need for species-level discrimination and the ability to withstand diverse and extreme storage conditions such as heat, humidity and the lack of a cold chain. Finally, the document suggests a cost per test of $1–5, with a testing device costing ≤ $2000. The goals of the TPP overlap with the WHO-coined acronym REASSURED which is a general guideline for a successful diagnostic test: Real-time connectivity, Ease of specimen collection, Affordable, Sensitive, Specific, User-friendly, Rapid and robust, Equipment-free and Deliverable to end-users (Otoo and Schlappi, 2022).

In this review, we discuss the shortcomings of existing CL diagnostics, as well as recent developments which may enable the development of a diagnostic tool matching the benchmarks in the WHO TPP by the 2030 deadline.

## Aetiology and pathogenesis of CL

**Figure 2.**
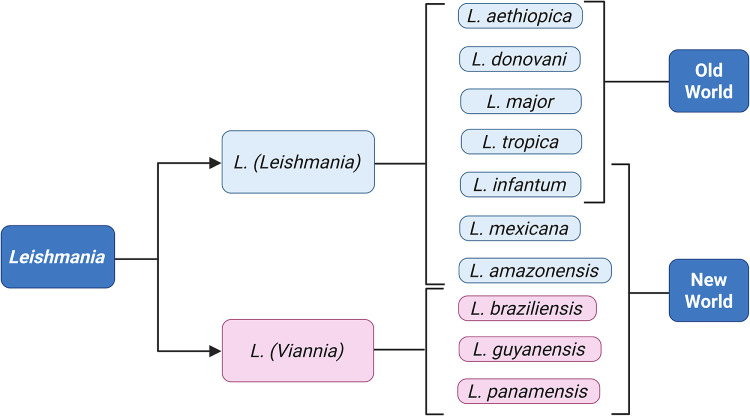
The most common *Leishmania* species causing disease in humans. Generated using BioRender. Figure 2 long description.

Human leishmaniasis is caused by 20 different species of *Leishmania* (Akhoundi et al. 2016); the most common disease-causing ones are summarised in Figure 2. Old World CL is caused by species of the *Leishmania (Leishmania)* subgenus, namely *Leishmania major, Leishmania tropica, Leishmania aethiopica* and less frequently, *Leishmania infantum* and *Leishmania donovani* (Akhoundi et al. 2016; Gurel et al. 2020). New World CL and MCL are caused by *L. (Viannia)* species such as *Leishmania braziliensis, Leishmania guyanensis* and *Leishmania panamensis*, although *L. (Leishmania)* species such as *L. infantum, L. mexicana* and *Leishmania amazonensis* are also endemic in the region (Akhoundi et al. 2016; Gurel et al. 2020).

The disease is transmitted to the mammalian host via a bite from the insect sandfly vector (Akhoundi et al. 2016). The simplest clinical presentation is localised CL: individual self-healing lesions at the site of infection (Aronson et al. 2016). *Leishmania major* infections typically follow this pattern, with lesions usually healing after 2–6 months (Auwera G and Dujardin, 2015). In contrast, *L. tropica* lesions typically take 6–15 months to heal (Aronson et al. 2016). *Leishmania major* CL was first distinguished by its ‘wet rural’ lesions, as opposed to the ‘dry urban’ lesions caused by *L. tropica* (Akhoundi et al. 2016).

*Leishmania tropica* is traditionally considered anthroponotic (human to vector to human transmission), as opposed to most other *Leishmania* infections which are zoonotic (animal to vector to human transmission) (Gurel et al. 2020; Reimão et al. 2020). This urban, anthroponotic transmission pattern makes *L. tropica* a great threat in times of large population displacement, such as during the Syrian civil war or the recent regime change in Afghanistan (Kim et al. 2025; Rahimi et al. 2025). *L. tropica* is also considered more genetically diverse than *L. major* (Schwenkenbecher et al. 2006; Pratlong et al. 2009; Iantorno et al. 2017).

*Leishmania aethiopica* is a CL-causing species found exclusively in Ethiopia and neighbouring countries (Blaizot et al. 2024). Ethiopia is 1 of the 10 most affected countries for CL, with an estimated 20 000 to 50 000 cases annually (Taye et al. 2024). Although there is some discrepancy in recent studies about the level of genetic diversity, it was indicated that *L. aethiopica* is more diverse than expected given its limited range (Hadermann et al. 2023; Yizengaw et al. 2024). This diversity may account for the poor performance of commercial diagnostic tests reported in Ethiopia (Van Henten et al. 2022; Taye et al. 2024).

As mentioned above, *L. infantum* and *L. donovani* can cause CL despite being primarily viscerotropic (Akhoundi et al. 2016). *L. donovani* can also cause post-kala-azar dermal leishmaniasis (PKDL) where, a visceral disease is followed by dermal symptoms (Reimão et al. 2020). These atypical disease forms are a challenge to VL elimination efforts as they create an additional reservoir of *L. donovani* (Jain et al. 2024a; Bhattarai et al. 2025). Other complex forms of CL include leishmaniasis recidivans caused by *L. tropica* and diffuse CL (DCL) and MCL caused by *L. aethiopica* (Krayter et al. 2014, 2015; Aronson et al. 2016; Atnafu et al. 2024). A recent study in Ethiopia found a lack of consensus in how different physicians classify DCL and MCL (van Henten et al. 2025). This also poses a challenge as systemic treatment is recommended for these atypical CL forms, as opposed to localised CL.

New World CL species such as *L. amazonensis, L. braziliensis, L. guyanensis* and *L. panamensis* also present a complex picture as they can cause both CL and MCL, while only *Leishmania mexicana* causes CL exclusively (Mann et al. 2021). *L. infantum*, often named *Leishmania chagasi* in the American context, also causes New World CL and VL but not MCL (de Vries and Schallig, 2022).

Whilst self-healing lesions can be left untreated, incorrect diagnosis and treatment can result in scarring and disfigurement, as well as opportunistic secondary infections (Akuffo et al. 2023; Aminizadeh et al. 2024; Jaimes et al. 2024; Al-Alousy and Al-Nasiri, 2025). There are no universally efficacious treatments for CL as some *Leishmania* species in certain geographical settings show drug resistance (Aronson et al. 2016). For example, antimonial resistance in *L. aethiopica* and *L. tropica* infections has been reported in Ethiopia, Iran and Pakistan (Kämink et al. 2021; Solomon et al. 2022; Zewdu et al. 2022). Drug resistance has also been reported in *L. mexicana* and *L. braziliensis* (Ahmed et al. 2020; Gonzalez-Garcia et al. 2025; Ko et al. 2025; Moreno-Rodríguez et al. 2025).

**Figure 3.**
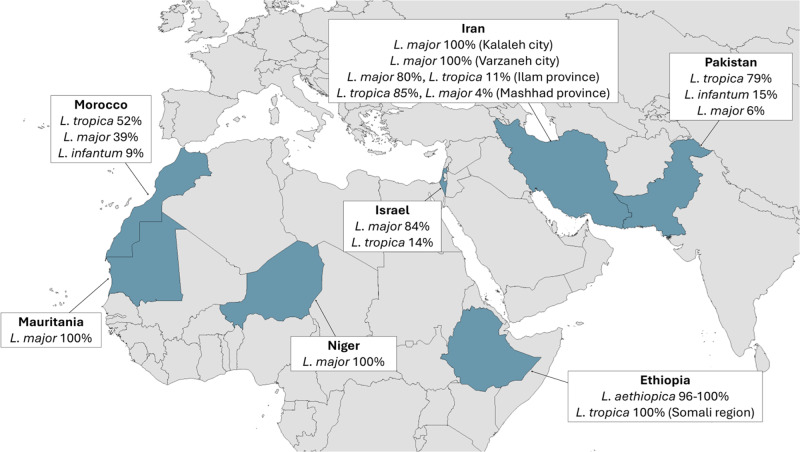
Countries where OWCL species prevalence was reported in the years 2023–2025. Figure 3 long description.

For the reasons described above, species identification is crucial to the effective treatment and control of CL. Unfortunately, due to the costly and time-consuming nature of species typing, data on the prevalence of individual CL *Leishmania* species remains limited and sporadic (Figure 3). A study in the Khyber Pakhtunkhwa province in Northern Pakistan found that 90% of CL cases were caused by *L. tropica*, despite the initial clinical examination finding that 73% of the lesions were dry (presumed *L. tropica*) and 27% were wet (presumed *L. major*) (Ullah et al. 2024). This indicated that a majority of the 118 patients who had wet lesions were actually infected with *L. tropica*, which is an atypical presentation and demonstrates that clinical symptoms alone are insufficient for accurate diagnosis. A nationwide study found that 79% of CL cases were *L. tropica*, 15% were *L. infantum* and 6% were *L. major*, confirming that *L. tropica* is the main driver of CL in Pakistan (Naz et al. 2024). In neighbouring Afghanistan *L. tropica* has historically accounted for the majority of cases, although more recent data on this is not available (Rahimi et al. 2025). In Israel, Mauritania and Niger, the situation is reversed, with 84–100% of cases caused by *L. major* (Avni et al. 2024; Blaizot et al. 2025; El Moctar et al. 2025). In Iran the dominant species can be *L. major* or *L. tropica*, depending on the region (Zeinali et al. 2023; Hayatolgheib-Moghadam et al. 2025; Marvi-Moghadam et al. 2025). In Morocco 52% of cases were caused by *L. tropica*, 39% *L. major* and 9% *L. infantum* (Baghad et al. 2025). Recent studies in Ethiopia found *L. aethiopica* in 96% to 100% of samples (Amare et al. 2023; Tesfaye et al. 2025). Interestingly, a study of a recent CL outbreak in the conflict-stricken Somali region of Ethiopia, reported 100% of samples as *L. tropica* (Abera et al. 2025). However, this finding should be treated with a note of caution as only 18 of the 1050 patients in the study were sampled for species typing. Nevertheless, in 99% of all 1050 patients the lesions examined were wet, unlike the typically dry lesions caused by *L. tropica* or *L. aethiopica*. The outbreak was also characterised by an unusually high number of lesions per patient, with 90% of patients having seven or more lesions.

Equivalent data for New World CL is also sparse. A study in Roraima state in Brazil found *L. braziliensis* in 32% of samples, *L. amazonensis* in 17% of samples and *L. panamensis* in 13% of samples (de Almeida et al. 2021). A further 6 species were found in 14% of samples and in 24% of samples the species could not be identified. A study in Colombia found *L. panamensis* in 77% of samples, *L. guyanensis* in 19% of samples and *L. braziliensis* in 4% of samples (Hoyos et al. 2022). In a study in Bolivia, 50% of CL samples were identified as *L. braziliensis* and 50% as subpopulation called *L. braziliensis* outlier (Torrico et al. 2022). Finally, a study in Panama found *L. panamensis* in 59% of samples and *L. guyanensis* in 41% of samples, although not all results could be confirmed after further testing (Reina et al. 2022).

## Traditional diagnostic methods

As discussed, CL lesions can be visually classified as wet or dry, with additional criteria such as nodular, popular, plaque or ulcerated (Naz et al. 2024; Ullah et al. 2024). As mentioned, these characteristics correlate with the infecting species, but not reliably enough for accurate diagnosis (Naz et al. 2024; Ullah et al. 2024). CL can be confirmed by microscopic examination of a direct smear from the lesion (de Vries and Schallig, 2022). A Giemsa stain allows the *Leishmania* amastigote forms (the mammalian stage) to be identified, however is not species specific, requires technical expertise and has limited sensitivity (50–70%) (Aronson *et al.* 2016; Reimão et al. 2020; de Vries and Schallig, 2022). Parasite isolation by culture can improve sensitivity and allows further analytical tests such as PCR, but it prolongs the diagnosis time, is highly technical and liable to contamination (Reimão et al. 2020).

Patient sampling methods include scraping, brushing, swabbing and aspiration (Reimão et al. 2020). The choice of technique is influenced by cosmetic significance and patient comfort (Aronson et al. 2016). A recent study compared methods for cutaneous lesions – skin slit, dental broach, tape dis and microbiopsy (van Henten et al. 2024). The skin slit had the highest self-reported pain score with a median of 6/10 compared to dental broach (4/10) and tape disc and microbiopsy (both 1/10). Tape disc and microbiopsy had poorer sensitivity (69–83%), compared to the dental broach (96%) and skin slit (95%). Another study comparing lancet scraping, filter paper imprints and microbiopsy samples for kDNA-PCR found that lancet scraping was the most sensitive method (99%) compared to filter paper imprints (95–96%) and microbiopsy (93–94%) (De Los Santos et al. 2024).

## Serological and immunological tests

Serological and immunological tests like the Leishmanin Skin Test have been in use for almost 100 years (Dey et al. 2023). The direct agglutination test (DAT), a later development where killed *Leishmania* promastigotes are mixed with patient blood or serum, was found to have 95% sensitivity and 95% specificity for VL in a recent meta-analysis (Roberts et al. 2023). Commercial ELISA tests for VL are also available. A recent study compared NovaLisa (Novatec), Bordier (Bordier Affinity), Ridascreen (R-Biopharm) and Vircell (Vircell) (Lévêque et al. 2020). The tests showed 81–94% sensitivity and 96–100% specificity, although they take 2.5–3.5 hours to perform. The four commercial ELISAs also performed poorly on samples from immunocompromised patients, with 15–42% false negative results. A separate study reported 96% sensitivity and 81% specificity for NovaLisa (Rune Stensvold et al. 2019).

Despite the promising performance with VL samples, these kinds of tests are not recommended for CL as the infection does not produce a strong humoral response (de Vries and Schallig, 2022). Additionally, serological tests do not distinguish past and active infection, and cross-reactivity has been shown with Chagas Disease, caused by the closely related *Trypanosoma cruzi*, but also with leprosy, tuberculosis, typhoid fever and malaria (Aronson et al. 2016). Despite this, a meta-analysis of patents for *Leishmania* diagnostics in the years 2010–2022 found that 63 of the patents were based on immunological methods whereas only 33 were based on molecular methods (de Avelar et al. 2023). Among those 33, only six were tested against *L. tropica* and none against *L. aethiopica* respectively.

## PCR-based methods

PCR is an established laboratory technique for amplifying a specific DNA sequence with end-point or real-time detection (qPCR) (Auwera G and Dujardin, 2015). Downstream analyses, such as restriction fragment length polymorphism (RFLP) or nested PCR can improve specificity and sensitivity (Akhoundi et al. 2017). Both approaches have been used for species identification in *Leishmania* spp. (Ashraf et al. 2025; Mohammadi Manesh et al. 2025). Melting curve analyses can also be used in this way, as the melting temperature of double-stranded DNA is sequence-specific. The technique has been used to differentiate PCR products from *L. major, L. tropica* and *L. donovani* (Azam et al. 2024).

Diagnosis of leishmaniasis by PCR-based approaches has already been reviewed extensively (Auwera G and Dujardin, 2015; Akhoundi et al. 2017; Reimão et al. 2020; Thakur et al. 2020; Nharwal et al. 2025). One of the most common targets is the ribosomal gene array, particularly the 18S subunit gene and the intergenic spacer ITS1 (Boulal et al. 2025; Mughal et al. 2025). The kDNA minicircle, a DNA fragment of approximately 800bp found in the kinetoplast has also been used (Roozbehani et al. 2025). The kinetoplast is a DNA-containing structure in the mitochondria that is unique to the Kinetoplastida, such as *Leishmania* and *Trypanosoma* spp. and has long been favoured as a PCR target due to its very high copy number reaching as many as 10 000 copies (Noyes et al. 1998). The kinetoplast also comprises larger fragments called maxicircles containing the cytochrome b gene, which is another target (Mughal et al. 2025). Recently, cytochrome c oxidase I, also located on the maxicircle, has been identified as a new alternative (Mata-Somarribas et al. 2024). Others include cysteine protease B (cpb), glycoprotein GP63, heat-shock protein 70 (hsp70) and 7SL-RNA (Chaouch et al. 2013; Hosseini-Safa et al. 2018; Azam et al. 2024).

A recent meta-analysis covering the years 2011–2022 found a pooled sensitivity of 91% and pooled specificity of 98% for qPCR (Rihs et al. 2025b). An update by the same authors found that sensitivity and specificity of a qPCR approach against VL was 90% and 99.6% overall, but 86% and 100% when non-invasive sampling was used (Rihs et al. 2025a). The authors cite urine samples as a non-invasive alternative to blood and bone marrow samples which are typically used in the diagnosis of VL. For CL, the sensitivity and specificity were 87% and 87% when invasive sampling was used, but only 70% and 96% using non-invasive sampling. Urine samples and lesion swabs, imprints and aspirates were classified as non-invasive methods as opposed to lesion biopsies, scrapings and smears.

The authors note that for VL samples the sensitivities reported in the original papers were often calculated using serological tests as the reference (Touria et al. 2018; Iatta et al. 2021; Pereira et al. 2021). For CL samples, because of the weaker humoral response the reference techniques used were microscopy, immunohistochemistry or a second molecular test such as conventional PCR (Vink et al. 2018; Chaouch et al. 2019; Silgado et al. 2021). Therefore, while the gene targets are often the same, comparisons in sensitivity between VL and CL diagnostic assays should be made with caution due to these discrepancies.

Despite the promising results of various PCR-based assays in the laboratory, their deployment in the field is hampered by the requirement for expensive laboratory facilities and trained technical staff (Thakur et al. 2020). Furthermore, the reduced sensitivity to CL compared to VL, particularly when non-invasive sampling is used, is a notable drawback.

In an attempt to overcome the technical barrier, miniature PCR devices have been developed. Palm PCR, a miniature battery-powered PCR incubator, was able to detect *L. tropica, L. infantum* and *L. major* with a limit of detection of 40 pg, 4 pg and 0.4 pg, respectively (Bel Hadj Ali et al. 2023). The authors created a duplex assay which could distinguish *L. major, L. tropica* and *L. infantum* and by coupling the PCR reaction to a lateral flow test (LFT) the results could be visualised in under 30 minutes. One drawback of the study was the lack of clinical samples tested. An alternative device, the miniPCR thermocycler, has been used to detect *L. panamensis* (Castellanos-Gonzalez et al. 2023). The authors used the Prismo Mirage smartphone app to quantify the fluorescent signal obtained with the miniPCR transilluminator. Amplification by miniPCR had equal sensitivity (100%) to a reference qPCR assay when tested on samples from Colombia and Peru, although the time to amplification was not recorded. The miniPCR has also been used in combination with an LFT against VL samples from Ethiopia (Hagos et al. 2024). The test showed 96% sensitivity and 99% specificity relative to qPCR, giving results in just over an hour. The same assay was tested on VL samples from Spain and East Africa (van Dijk et al. 2024). In Spain, miniPCR had 96% sensitivity and 97% specificity, while in East Africa it had 88% sensitivity and 100% specificity when compared to nested PCR.

A few commercial PCR kits have been developed for the diagnosis of leishmaniasis; a recent study compared STAT-NAT (Sentinel), Real-TM (Sacace) and VIASURE (CerTest) (Arnau et al. 2023). Whilst Real-TM requires −20^o^C storage, STAT-NAT and VIASURE both allow storage at room temperature. When tested against a range of species, VIASURE and Real-TM detected both Old World and New World species, while STAT-NAT could only detect Old World species and was therefore excluded from further analysis. When tested against clinical samples, VIASURE had 82% sensitivity and 100% specificity compared to Real-TM.

Another interesting development has been adaptive PCR, where fluorescently labelled L-DNA probes are used to model primer annealing efficiency, which allows the PCR conditions to be adjusted in real time and the amplification rate to be increased accordingly. A recent study using this technique was able to achieve 20 cycles of amplification in under 15 minutes due to the reduced cycle duration (Spurlock et al. 2025). An alternative method is microfluidic qPCR, such as that developed by GeneSoC, which was shown to be able to achieve 50 cycles in 15 minutes (Mizushina et al. 2025). These findings, which have not been applied to *Leishmania* yet, could enable the development of a cheap and rapid PCR test for leishmaniasis for use in the field.

## Isothermal amplification

**Figure 4.**
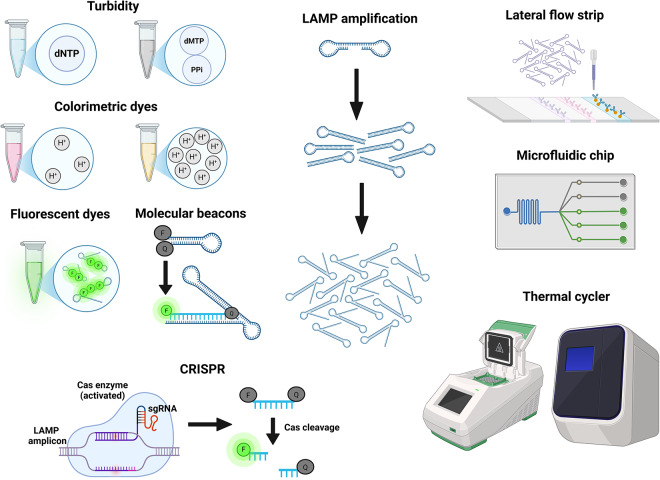
A visual summary of common approaches and devices used for the detection of LAMP amplification. Generated using BioRender. Figure 4 long description.

Loop-mediated Isothermal Amplification (LAMP) was developed in 2000 (Notomi et al. 2000) and uses a thermostable Bst Polymerase with strand displacement activity. This, together with the use of a second outer pair of primers, facilitates strand separation without the high temperature melting step used in PCR and allows the whole reaction to take place at the same temperature (isothermal). A subsequent adaptation incorporating a third primer pair increases the amplification speed (Nagamine et al. 2002). Various approaches for monitoring the LAMP reaction, such as colorimetric or fluorescent dyes and turbidity caused by the byproduct pyrophosphate, are available (Figure 4) (Poirier et al. 2023; Jin et al. 2024; Quero et al. 2025). The assay can also be coupled with LFTs for ease of detection (Sanmoung et al. 2023). LAMP-LFT has not been used in the diagnosis of leishmaniasis, but similar approaches combining LFTs with PCR and RPA (Recombinase Polymerase Amplification, an alternative isothermal technique) have been reported (Cossio et al. 2021; Hagos et al. 2024; Nawattanapaibool et al. 2024; van Dijk et al. 2024).

The simultaneous use of three primer pairs provides a high degree of specificity. Indeed, LAMP assays have been used to detect point mutations conferring drug resistance (Sanmoung et al. 2023). Specificity can be further improved by detection with molecular beacons. A molecular beacon is a fluorophore in a quenched form, either as dsDNA or hairpin ssDNA. The fluorophore is released from the quencher when the beacon binds a specific sequence on the LAMP amplicon (Xue et al. 2024). This approach allows multiplexing as different fluorophores can be used with each beacon (Sherrill-Mix et al. 2021). The technique has not been applied to *Leishmania spp.*, but it has been tested with *Trypanosoma brucei* and *Schistosoma mansoni* (Wan et al. 2017; Crego-Vicente et al. 2023).

An alternative approach for increasing specificity is integration with CRISPR/Cas9 (Liang et al. 2024). Briefly, the Cas endonuclease becomes activated by binding a target sequence on the LAMP amplicon with the help of a guide RNA and, subsequently, cleaves a short reporter DNA fragment to release the fluorophore from its quencher. The specific combination of LAMP and CRISPR has not been applied to leishmaniasis yet, although other studies have combined CRISPR with PCR and with RPA (Dueñas et al. 2022; Yang et al. 2024). CRISPR-assisted LAMP has also been used to diagnose toxoplasmosis, demonstrating its feasibility in parasitic infections (Liang et al. 2024).

In summary, the high sensitivity and specificity due to the use of three primer pairs, the rapid amplification time and the simple technical requirement of an isothermal heat source, make LAMP a very promising technique for a low-cost, species-specific, field diagnostic, such as that required for CL. Multiple LAMP assays for the diagnosis of leishmaniasis have been published and reviewed previously (Auwera G and Dujardin, 2015; Akhoundi et al. 2017; Nzelu et al. 2019; Reimão et al. 2020; Thakur et al. 2020; de Vries and Schallig, 2022; Nharwal et al. 2025). The gene targets used are typically the same as those for PCR as they share the same characteristics, namely interspecies variation and high copy number.

The most recent novel assay targeting hsp70 achieved 100 fg sensitivity (Soares et al. 2024). This is a useful benchmark as it is the approximate amount of DNA in a single *Leishmania* parasite cell (Soares et al. 2024). The study found no cross-reactivity to *T. cruzi* and *T. brucei*, which had been observed with other LAMP assays for *Leishmania* spp. However, the reaction time of 60 minutes is a major drawback. Furthermore, the test is not species specific and the study focused on New World *Leishmania* spp. such as *L. braziliensis*, and whilst it includes some Old World species like *L. major* and *L. infantum*, it does not include *L. tropica* and *L. aethiopica*.

Diagnostic assays for leishmaniasis often focus on species for which effective diagnostic tests are already available, like *L. donovani* and *L. infantum*, while species which cause a substantial part of the CL burden like *L. tropica* and *L. aethiopica* remain overlooked. These choices are undoubtedly informed by existing knowledge of endemic species in the regions where researchers are based, but this is by no means fixed in stone. Recent years have shown that *Leishmania* species can be rapidly introduced into non-endemic countries, either by human migration or by animal reservoirs (Azami-Conesa et al. 2024; Kim et al. 2025).

To date, only three studies have designed LAMP primers to discriminate *Leishmania* at the species level. The first used primers targeting the kDNA minicircle of *L. donovani* with a limit of detection of 1 fg and no cross-reactivity against *L. infantum, L. major* or *L. tropica* (Verma et al. 2013). A further study used primers targeting CPB to distinguish *L. major* and *L. tropica* with a limit of detection of 20 fg and 200 fg, respectively (Chaouch et al. 2019). Most recently, one study used primers targeting kDNA minicircle of *L. tropica* with a limit of detection of 1 fg and no cross-reactivity for *L. major* or *L. infantum* (Taslimi et al. 2023). All of these assays demonstrated detection levels of two cell equivalents or less.

RPA is an alternative isothermal technique which requires lower temperatures (37^o^C–42^o^C) than LAMP (Wu et al. 2023). However, the technique requires a recombinase and a single-strand DNA-binding protein in addition to the DNA polymerase which may be a challenge with respect to developing a lyophilisation protocol. Nevertheless, some recent studies have successfully used RPA to detect *Leishmania* spp. (Danthanarayana et al. 2023; Ghosh et al. 2023; Roy et al. 2023; Wu et al. 2023; Bel Hadj Ali et al. 2024; Kobialka et al. 2024; Wiggins et al. 2024).

## Sequencing-based approaches

Targeted and whole-genome DNA sequencing (WGS) has promising uses in diagnostics. Species typing of a CL infection by targeted sequencing using the Oxford Nanopore MinION platform has been reported and the technique can show evidence of mixed or hybrid infection (Imai et al. 2018; Maia de Souza et al. 2022; Patiño et al. 2023; Jaimes et al. 2024). The platform has likewise been used in the detection and genetic typing of other protozoan parasites such as *Trypanosoma, Toxoplasma* and *Plasmodium* spp. (Cruz-Saavedra et al. 2024; Huggins et al. 2024; Koutsogiannis and Denny, 2024). While targeted sequencing can provide an indication of mixed or hybrid infection, WGS is required to distinguish between the two, as true hybrids are marked by genome-wide changes in somy and allele frequencies (Lypaczewski et al. 2022; Van den Broeck et al. 2023). Monitoring the emergence of hybrids is crucial as these can produce atypical forms of disease (Lypaczewski et al. 2022; Bruno et al. 2024).

WGS can also be used for surveillance, as was shown with *L. donovani* isolates in Nepal (Monsieurs et al. 2024). In the context of the current VL elimination program in Nepal, this can be used to distinguish VL cases caused by strains introduced from external sources from cases caused by endemic strains. Finally, generating new genome assemblies can provide more data on sequences and copy numbers for diagnostic targets such as hsp70, 18S and GP63 (Alonso et al. 2016; Bussotti et al. 2018).

## Point-of-care and rapid diagnostic tests

Immunochromatographic LFTs (ICT) are widely used in the detection of VL and include IT LEISH (Bio-Rad), TruQuick (Meridian) and Kalazar Detect (InBios). In two recent studies on VL samples from Brazil and the Mediterranean, the sensitivity of these tests was 85–96% while specificity was 96–99% (Freire et al. 2019; Lévêque et al. 2020). However, the sensitivity with samples from immunocompromised patients was poorer, dropping to as little as 47%.

An alternative ICT specific for CL is CL Detect (InBios), targeting the peroxidoxin antigen. A recent meta-analysis of studies testing CL Detect found 68% pooled sensitivity and 94% pooled specificity (Gebremeskele et al. 2023). The authors noted that the lack of consistency in the reference diagnostic used explained some of the discrepancies. Studies where microscopy was used as the reference reported higher sensitivity (83%) than those which included the more sensitive PCR as reference (48%). One of the studies reviewed compared different sampling methods. Sampling by dental broach had 64% sensitivity and 92% specificity, while lancet scraping had 83% sensitivity and 78% specificity (Grogl et al. 2023). One study already discussed above also compared skin slit, dental broach, microbiopsy and tape disc (van Henten et al. 2024). Skin slit and dental broach performed similarly (95–96% sensitivity), tape disc and microbiopsy samples were considerably poorer (69–83% sensitivity). Notably, when skin slit PCR was used as the reference, the other three sampling methods had very low specificity (27–73%) compared to when a composite reference was used (85–100%). As multiple authors have noted that the sensitivity and specificity of a test can be distorted depending on which technique is used as a reference, more effort is needed in future studies to ensure consistency (Van Henten et al. 2022; Gebremeskele et al. 2023). While the TPP targets for sensitivity and specificity are expressed relative to microscopy, PCR is a more sensitive technique; the ideal approach is to use both as a composite reference, making it easier to compare between studies.

Loopamp is a LAMP-based test kit developed by Eiken Chemical, a spin-out company created by the authors of the LAMP technique (Feddema et al. 2024). This multiplex assay, with genus-specific primers targeting both the 18S rRNA gene and the kDNA minicircle, is lyophilised, allowing storage at room temperature. A recent meta-analysis found Loopamp had a pooled sensitivity of 96% for VL and 93% for CL, and 99% and 87% specificity, respectively (Taye et al. 2025). This makes Loopamp the best-performing diagnostic test for CL to date and highlights the potential of LAMP as the most optimal technique for fulfilling the requirements of the TPP (Table 1). Also promisingly, sensitivity and specificity were 96% and 99% when crude Boil&Spin DNA extraction was used, compared to 97% and 99% with the commercial Qiagen extraction kit. As this was tested in VL samples only, caution is needed. Additionally, as there is a variety of sampling methods for CL lesions, with no consensus on which one is most effective, much work remains to be done in this area.

**Table 1.** The performance of various diagnostic tests for leishmaniasis as calculated by recent meta-analyses, compared to the WHO target product profile Table 1 long description.

Test name	Sensitivity (%)	Specificity (%)	References
**Target product profile**	**90**	**95–100**	(Cruz et al. 2019)
**Direct agglutination test (DAT)**			
VL	95	95	Roberts et al. (2023)
**CL detect (InBios)**			
CL	68	94	Gebremeskele et al. (2023)
**qPCR**	–	–	–
VL	90	99.6	Rihs et al. (2025b); Rihs et al. (2025a)
CL	87	87	–
**Loopamp (Eiken chemical)**	–	–	–
VL Qiagen DNA extraction kit	97	99	Taye et al. (2025)
VL crude DNA extraction	96	99	–
CL Qiagen DNA extraction kit	93	87	–

Other improvements are also needed. Loopamp showed only 48% sensitivity on CL samples in Ethiopia (Taye et al. 2024). One drawback of that study was that tape discs were used for sample collection which have been shown elsewhere to be less sensitive than other sampling methods (van Henten et al. 2024). CL Detect also had its poorest result in Ethiopia, showing 23–31% sensitivity (Van Henten et al. 2022). This indicates a broader problem with the genetic diversity of *L. aethiopica* being unaccounted for when these tests are being designed. Furthermore, there are no published trials of Loopamp in Pakistan and Syria, which have borne a large part of the CL burden in recent years. Testing clinical samples from a wide range of geographical locations is crucial. A recent study on diagnostic PCR assays for soil-transmitted helminth infections demonstrated that global genetic differences in primer binding sites can have a detrimental impact on assay sensitivity as these assays are often based on reference strains of the pathogen (Papaiakovou et al. 2025).

Finally, despite the apparent efficacy of Loopamp outside of Ethiopia, it is only a genus-specific test. At the time of writing, there is no species-specific RDT for *Leishmania spp*. This feeds into a vicious cycle: because species typing in *Leishmania* spp. is time-consuming and requires laboratory facilities and trained staff, data on individual species prevalence is sparse, which hinders the development of future diagnostic tests. A handful of promising species-specific isothermal NAATs have been published, but further work needs to be done validating these across a variety of strains and diverse clinical samples and new assays need to be designed to cover species and geographical foci that have been previously overlooked.

## Isothermal amplification devices

The earliest specialised device for isothermal amplification was the Genie I (Optigene), first used in a study in 2010 (Tomlinson et al. 2010). The miniaturised real-time fluorimeter was in its third iteration (the Genie III) by the time it was trialled in the COVID-19 pandemic (Fowler et al. 2021). Although prices for the Genie are not publicly available, they can be found through secondary retailers as costing $10 000–$21 000. This is cheaper than traditional real-time PCR thermocyclers, but the price still exceeds the $2000 TPP target price (Cruz et al. 2019). More recently, Eiken Chemical have developed the LF-160, an isothermal incubator with a UV transilluminator for qualitative visual detection, costing $2000 (Sohn et al. 2019).

Most trials of Loopamp used the LF-160 (Mukhtar et al. 2018; Hagos et al. 2021; Hossain et al. 2021). Only one study tested Loopamp in three different devices – the LF-160, the Genie III and ESE-Quant (Qiagen) (Ibarra-Meneses et al. 2018). All three devices showed the same sensitivity, although amplification in the Genie III was much faster and matched the amplification times of the qPCR assay more closely than ESE-Quant. As the LF-160 incubator does not have a real-time function there was no equivalent data to compare.

**Figure 5.**
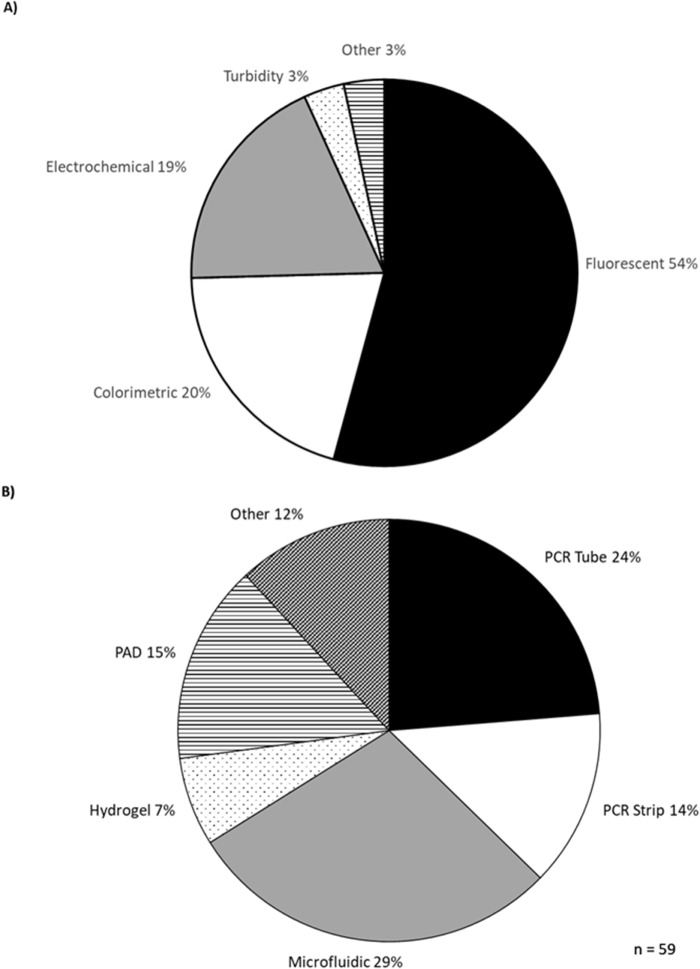
Detection methods (A) and sample formats (B) used in new isothermal diagnostic devices. Figure 5 long description.

**Figure 6.**
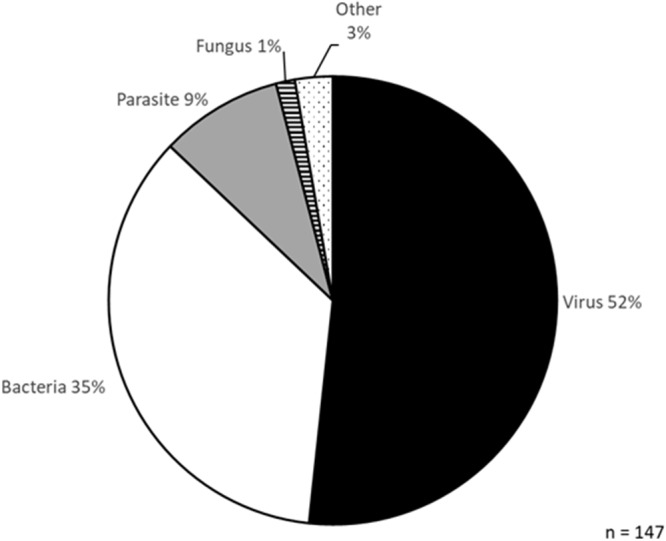
Classes of pathogens tested in new isothermal diagnostic devices. Figure 6 long description.

The development of isothermal diagnostic devices accelerated with the COVID-19 pandemic and many of these have been reviewed elsewhere (Das et al. 2022; Narasimhan et al. 2023). A selection of devices published since is summarised in the Supplementary Information. Most studies use fluorescent detection (54%), followed by colorimetric (20%) and electrochemical (19%) (Figure 5). Most devices were designed for PCR strips (14%) or individual PCR tubes (24%) (Figure 6). Microfluidic chips were the next most common format, being used in 29% of studies. Microfluidic devices are a promising development in diagnostics but they need to be custom-made for each application, while PCR tubes have a cheap, simple and uniform design, which provides versatility and is an advantage where low cost and ease of use are the priority.

Only 20 (34%) of the studies listed a manufacturing cost for their device; of those, 13 (22%) were cheaper than the mini-PCR thermocycler, which costs $600 (Castellanos-Gonzalez et al. 2023). Crucially, all but one of the devices with a listed cost met the TPP target of $2000 or less.

Notably, the listed devices were tested on a range of different species, primarily viruses (52%) and bacteria (35%), with parasites accounting for only 9% (Figure 6). Pathogens causing NTDs accounted for 5% of the species tested. Only two studies focused on *Leishmania* spp. specifically. The first used fluorometric LAMP to detect 0.1 pg of *L. donovani* DNA (Puri et al. 2021). The other study used electrochemical sensing coupled to RPA, detecting 10 copies/µl (<1 pg) of *L. braziliensis* DNA (Wu et al. 2023). Therefore, more work needs to be done testing these new devices in the diagnosis of CL and other NTDs where they are desperately needed.

## Artificial intelligence

More recently, machine learning has also been tested for visual classification of CL lesions. Promisingly, studies in Brazil, Cameroon, and Colombia showed 93–96% accuracy, although a study in Libya showed only 70% accuracy (Arce-Lopera et al. 2021; Steyve et al. 2022; Mohamed Noureldeen et al. 2023; Leal et al. 2024). Whether this is due to species-specific differences in lesion appearance, differences in skin tone, or other discrepancies in the experimental approach is currently unknown. Notably, only the Brazilian study took the causative species used in the training set into account, which the authors identified as *L. braziliensis, L. amazonensis* and *L. guyanensis* (Leal et al. 2024). Therefore, more work remains to make sure that these approaches have high accuracy in a range of different endemic settings. In a different approach, a study in Iran tested three different algorithms and found that they could distinguish CL lesions which were responsive or non-responsive to treatment with 56–77% accuracy (Bamorovat et al. 2025). AI has also been used to visually diagnose other cutaneous NTDs – Buruli ulcer, leprosy, scabies, mycetoma and yaws (Yotsu et al. 2023). Buruli ulcer and scabies were correctly diagnosed with 88% sensitivity and yaws was correctly diagnosed with 79% sensitivity. However, specificity was very poor. Mycetoma was misdiagnosed as Buruli ulcer in 41% of cases, leprosy was misdiagnosed as scabies in 64% of cases and scabies was misdiagnosed as yaws in 14% of cases. Leprosy and mycetoma were correctly diagnosed in only 13% and 0% of cases, respectively.

AI has also been deployed in the detection of *Leishmania* spp. parasites by microscopy, with 73% to 99% accuracy (Abdelmula et al. 2024; Contreras-Ramírez et al. 2024; Kumar et al. 2024; Sadeghi et al. 2024; Tekle et al. 2024; Gadri et al. 2025). This represents a rapid improvement as a study from 2022 found 60% accuracy for infected macrophages (Zare et al. 2022). Although this approach promises to substantially reduce workload and minimise human error, it does not eliminate many of the previously discussed shortcomings of microscopy-based diagnostic techniques. In fact, only one of the studies cited used a smartphone camera to capture the images from the microscope, while the others used high resolution digital cameras (Gadri et al. 2025). Promisingly, the use of a smartphone camera did not compromise accuracy, which was 97%. Nevertheless, other issues such as the need for microscopy facilities and technical expertise in preparing the sample limit the applicability and relevance of these developments in a point-of-care setting.

## Telemedicine

The use of mobile apps and telemedicine to augment CL diagnosis is another emerging field. A recent review on the use of telemedicine in NTDs found that it increases the capacity of the healthcare system to track the disease (Salvador et al. 2025b). One of the case studies was an app developed in Colombia for the use of patients and healthcare professionals. The app made it easier to follow-up on patients’ disease progression, allowed the collection of data in the form of lesion photographs and reporting of adverse events and made patients more likely to adhere to treatment (Cossio et al. 2023). Another study in Brazil found a high rate of patient satisfaction in using telemedicine for a CL consultation, with one of the advantages listed as saving money on travel costs (Salvador et al. 2025a). The impact of the WHO’s own SkinNTDs app was recently studied in Cameroon (Moungui et al. 2025). The app scored highly on user satisfaction (3.3/4.5), quality of information (3.6/5) and impact on knowledge and diagnosis (3.9/5), although 57% of participants had limited experience with skin NTDs and reported encountering less than one case per month. In a comparable study conducted in Ghana and Kenya, where only 22% of participants reported encountering less than one skin NTD case per month, the app scored 3.8/5 on user satisfaction, 4/5 on quality of information and 4.5/5 on impact (Frej et al. 2022). While these reports are encouraging with regard to skin NTDs generally, it would be useful to see equivalent data in countries which are more affected by CL in particular. Another useful tool is Lesionia, an example of Digital Systems for Data Management, which allows clinicians and healthcare workers in different countries to log and record epidemiological data on CL (Harigua-Souiai et al. 2025).

The AI tools discussed above can be integrated with a telemedicine app to allow for a rapid triage step which can then lead to a referral for further testing. The use of AI image analysis tools in this way is already recommended by the WHO in the diagnosis of tuberculosis (World Health Organization, 2022). Remote diagnosis of CL by a mobile app was also successfully used on a small scale in Nepal during the COVID-19 pandemic (Parajuli and Prajapati, 2023).

## Conclusions

A number of advances have been made in recent years that bring the goals of the TPP closer to being realised. Most of the ingredients for a good diagnostic test for CL already exist, albeit scattered across different disciplines and research areas. The serological CL Detect RDT has achieved 93% specificity over various studies, but its sensitivity of 68% is far below the 95% target in the TPP (Gebremeskele et al. 2023). The isothermal Loopamp RDT improved on this substantially, with 93% sensitivity and 87% specificity (Taye et al. 2025). This is also an improvement on qPCR which was found to have 87% sensitivity and 87% specificity (Rihs et al. 2025a). However, both RDTs performed very poorly on samples from Ethiopia, which highlights the country as being particularly neglected in terms of readily available diagnostic tools (Van Henten et al. 2022; Taye et al. 2024).

The performance of the Loopamp test on crude VL samples indicates that a similar approach could be applied to CL samples, which would simplify sample preparation as outlined in the TPP. Additionally, a number of isothermal amplification devices matching the $2000 price target have now been developed. These could easily be integrated with a telemedicine app that uses AI-based visual lesion analysis as a triage step. This could have a dual effect of improving specificity by ruling out other lesion-causing dermatoses, but also meeting the goal of real-time connectivity, also listed in the TPP. Finally, the test result and lesion photograph could be logged together into a global database, to be regularly updated with patient information about the duration of the disease and responsiveness to treatments. In order to complete the process within the timeframe of the 2030 Roadmap, a focused and concerted effort remains to bring all of these elements together into a well-validated and coherent diagnostic algorithm.

## Supporting information

10.1017/S0031182025101467.sm001 Jarzabek and Denny supplementary material Jarzabek and Denny supplementary material

## Supplementary material

The supplementary material for this article can be found at https://doi.org/10.1017/S0031182025101467.

## Figure 1 Long description

The highlighted countries include Afghanistan, Bahrain, Djibouti, Egypt, Iran, Iraq, Jordan, Kuwait, Lebanon, Libya, Morocco, Oman, Pakistan, Palestine, Qatar, Saudi Arabia, Somalia, Sudan, Syria, Tunisia, UAE and Yemen. These countries are shaded to distinguish them from other regions in the surrounding areas.

Navigate back to Figure 1.

## Figure 2 Long description

The diagram illustrates the distribution of Leishmania species between the Old World and New World. It begins with a blue box labeled 'Leishmania,' which branches into two subgenera: 'L. (Leishmania)' in a light blue box and 'L. (Viannia)' in a pink box. The 'L. (Leishmania)' subgenus further branches into species associated with the Old World, including 'L. aethiopica,' 'L. donovani,' 'L. major,' and 'L. tropica,' as well as species associated with the New World, such as 'L. infantum,' 'L. mexicana,' and 'L. amazonensis.' The 'L. (Viannia)' subgenus includes species 'L. braziliensis,' 'L. guyanensis,' and 'L. panamensis,' all associated with the New World. The Old World species are highlighted in a blue box, while the New World species are highlighted in a pink box.

Navigate back to Figure 2.

## Figure 3 Long description

A thematic distribution map of North Africa, the Middle East and South Asia, with north at the top. Several countries are visually highlighted while surrounding countries are shown without highlighting. White callout boxes with leader lines label the highlighted countries and list Leishmania species percentages. Morocco: L. tropica 52 percent, L. major 39 percent, L. infantum 9 percent. Mauritania: L. major 100 percent. Niger: L. major 100 percent. Israel: L. major 84 percent, L. tropica 14 percent. Iran (callout lists multiple locations): - L. major 100 percent (Kaleleh city) - L. major 100 percent (Varzaneh city) - L. major 80 percent, L. tropica 11 percent (Ilam province) - L. tropica 85 percent, L. major 4 percent (Mashhad province) Pakistan: L. tropica 79 percent, L. infantum 15 percent, L. major 6 percent. Ethiopia (callout lists two entries): - L. aethiopica 96–100 percent - L. tropica 100 percent (Somali region).

Navigate back to Figure 3.

## Figure 4 Long description

A clean schematic infographic with multiple labelled icons and arrows arranged in three vertical areas. Left area shows five labelled sections with small tube and symbol drawings. The headings read: Turbidity, Colorimetric dyes, Fluorescent dyes, Molecular beacons and CRISPR. The Turbidity section shows two tube drawings and a circular inset with the text dNTP and PPi. The Colorimetric dyes section shows three tube drawings and a circular inset with small circle symbols. The Fluorescent dyes section shows a tube drawing and a circular inset with a glowing circle. The Molecular beacons section shows a beacon drawing with a small glowing circle and a looped line. The CRISPR section shows the label CRISPR, a Cas enzyme label, an sgRNA label, a short bar-like target graphic and the labels Cas cleavage and LAMP. Centre area shows the heading LAMP amplification above a vertical sequence of looped line drawings connected by two downward arrows. Right area shows three labelled sections. The headings read: Lateral flow strip, Microfluidic chip and Thermal cycler. The Lateral flow strip section shows a strip-like rectangle with line markings and a small droplet icon above it. The Microfluidic chip section shows a rectangular chip diagram with a channel-like path leading to several circular nodes. The Thermal cycler section shows two instrument drawings, one with an open lid and one as a tall rectangular unit.

Navigate back to Figure 4.

## Figure 5 Long description

The image A showing a pie chart with the labels and values: Fluorescent 54 percent, Colorimetric 20 percent, Electrochemical 19 percent, Turbidity 3 percent, Other 3 percent. The image B showing a pie chart with the labels and values: PCR Tube 24 percent, PCR Strip 14 percent, Microfluidic 29 percent, Hydrogel 7 percent, PAD 15 percent, Other 12 percent. Text at the bottom right reads n equals 59.

Navigate back to Figure 5.

## Figure 6 Long description

Classes of pathogens tested in new isothermal diagnostic devices. A pie chart with slice labels showing: Virus 52 percent. Bacteria 35 percent. Parasite 9 percent. Fungus 1 percent. Other 3 percent. Text on the figure: n equals 147. Virus is the largest slice. Bacteria is the second largest slice. Parasite, Other and Fungus are smaller slices.

Navigate back to Figure 6.

## Table 1 Long description

The table summarizes sensitivity and specificity (percent) reported by recent meta-analyses for diagnostic tests for leishmaniasis, separated by visceral leishmaniasis (VL) and cutaneous leishmaniasis (CL), and compares them with a target benchmark of 90% sensitivity and 95–100% specificity. For the direct agglutination test (DAT) in VL, sensitivity and specificity are both 95%, meeting the benchmark. CL Detect (InBios) for CL has 68% sensitivity and 94% specificity, falling below both target thresholds. qPCR results are mixed: for VL, sensitivity is 90% and specificity 99.6% (meets the benchmark), while for CL, sensitivity and specificity are both 87% (below target). Loopamp (Eiken Chemical) shows high performance for VL with either Qiagen extraction (97% sensitivity, 99% specificity) or crude extraction (96% sensitivity, 99% specificity), but for CL with Qiagen extraction sensitivity is 93% and specificity 87%, missing the specificity target. Several cells are blank or marked with dashes, indicating missing or not reported estimates for some test–disease combinations, so comparisons are limited to the reported meta-analysis values.

Navigate back to Table 1.

## References

[ref1] Abdelmula AM, Mirzaei O, Güler E and Süer K (2024) Assessment of deep learning models for cutaneous Leishmania parasite diagnosis using microscopic images. *Diagnostics* 14(1), 12. 10.3390/diagnostics14010012 PMC10802189 38201321

[ref2] Abera A, Tadesse H, Beyene D, Geleta D, Belachew M, Djirata EA, Kinde S, Difabachew H, Bishaw T, Hassen MA, Gire A, Bore TM, Berhe BM, Habtetsion M, Tugga ZO, Eyelachew E, Sefer WB, Choukri K, Coppens J, Tadese G, Haile K, Bekele H, Kebede Z, van der Auwera G, Seife F, Abte M, Tollera G, Hailu M, Dujardin J-C, van Griensven J, Wolday D, Embiale W, Pareyn M and Tasew G (2025) Outbreak of cutaneous leishmaniasis amongst militia members in a non-endemic district under conflict in the lowlands of Somali Region caused by Leishmania tropica, Eastern Ethiopia. *PLOS Neglected Tropical Diseases* 19(7), e0013246. 10.1371/journal.pntd.0013246 40694583 PMC12324667

[ref3] Ahmed B, Raut B, Pauley A, Davidson JL, Yang S and Verma MS (2025) Development of a portable paper-based biosensor for the identification of genetically modified corn (Zea mays) and soybean (Glycine max). *Biosensors and Bioelectronics* 287. 10.1016/j.bios.2025.117690 40543168

[ref4] Ahmed H, Curtis CR, Tur-Gracia S, Olatunji TO, Carter KC and Williams RAM (2020) Drug combinations as effective anti-leishmanials against drug resistant: Leishmania mexicana. *RSC Medicinal Chemistry* 11(8), 905–912. 10.1039/d0md00101e 33479685 PMC7651635

[ref5] Akhoundi M, Downing T, Votýpka J, Kuhls K, Lukeš J, Cannet A, Ravel C, Marty P, Delaunay P, Kasbari M, Granouillac B, Gradoni L and Sereno D (2017) Leishmania infections: Molecular targets and diagnosis. *Molecular Aspects of Medicine* 57, 1–29. 10.1016/j.mam.2016.11.012 28159546

[ref6] Akhoundi M, Kuhls K, Cannet A, Votýpka J, Marty P, Delaunay P and Sereno D (2016) A historical overview of the classification, evolution, and dispersion of leishmania parasites and sandflies. *PLoS Neglected Tropical Diseases* 10(3), e0004349. 10.1371/journal.pntd.0004349 26937644 PMC4777430

[ref7] Akuffo RA, Sanchez C, Amanor I, Amedior JS, Kotey NK, Anto F, Azurago T, Ablordey A, Owusu-Antwi F, Beshah A, Amoako YA, Phillips RO, Wilson M, Asiedu K, Ruiz-Postigo JA, Moreno J and Mokni M (2023) Endemic infectious cutaneous ulcers syndrome in the Oti Region of Ghana: Study of cutaneous leishmaniasis, yaws and Haemophilus ducreyi cutaneous ulcers. *PLoS ONE* 18(9), e0292034. 10.1371/journal.pone.0292034 37756291 PMC10529585

[ref8] Al-Alousy NW and Al-Nasiri FS (2025) Bacterial infections associated with cutaneous leishmaniasis in Salah Al-Din province, Iraq. *Microbial Pathogenesis* 198, 107144. 10.1016/j.micpath.2024.107144 39579944

[ref9] Alonso G, Rastrojo A, López-Pérez S, Requena JM and Aguado B (2016) Resequencing and assembly of seven complex loci to improve the Leishmania major (Friedlin strain) reference genome. *Parasites and Vectors* 9(1), 74. 10.1186/s13071-016-1329-4 26857920 PMC4746890

[ref10] Amare GA, Mekonnen GG, Kassa M, Addisu A, Kendie DA, Tegegne B, Abera A, Tadesse D, Getahun S, Wondmagegn YM, Merdekios B, Asres MS, van Griensven J, Van der Auwera G, van Henten S and Pareyn M (2023) First report of cutaneous leishmaniasis caused by Leishmania donovani in Ethiopia. *Parasites and Vectors* 16(1), 457. 10.1186/s13071-023-06057-9 38104111 PMC10725588

[ref11] Aminizadeh S, Mohammadi-Ghalehbin B, Mohebali M, Hajjaran H, Zarei Z, Heidari Z, Akhondi B, Alizadeh Z and Aghaei J (2024) Emergence of coinfection with visceral Leishmania infantum in COVID-19 patients: A case‒control field study in an endemic area of visceral leishmaniasis in Iran. *BMC Infectious Diseases* 24(1), 1480. 10.1186/s12879-024-10363-7 39736578 PMC11686996

[ref12] Arce-Lopera CA, Diaz-Cely J and Quintero L (2021) Presumptive diagnosis of cutaneous Leishmaniasis. *Frontiers in Health Informatics* 10, 75. 10.30699/fhi.v10i1.278

[ref13] Arnau A, Abras A, Ballart C, Fernández-Arévalo A, Torrico MC, Tebar S, Llovet T, Gállego M and Muñoz C (2023) Evaluation of the diagnostic sensitivity of the VIASURE Leishmania real-time PCR detection kit prototype for the diagnosis of cutaneous and Visceral Leishmaniasis. *Transboundary and Emerging Diseases* 2023, 1–8. 10.1155/2023/1172087 PMC12017045 40303796

[ref14] Aronson N, Herwaldt BL, Libman M, Pearson R, Lopez-Velez R, Weina P, Carvalho EM, Ephros M, Jeronimo S and Magill A (2016) Diagnosis and treatment of Leishmaniasis: clinical practice guidelines by the Infectious Diseases Society of America (IDSA) and the American Society of Tropical Medicine and Hygiene (ASTMH). *Clinical Infectious Diseases* 63(12), E202–E264. 10.1093/cid/ciw670 27941151

[ref15] Ashraf A, Qadeer S, Bukhari UA, Zeb I, Rasool A, Salma U, Shaukat H, Shahzad R and Osmani AR (2025) Molecular profiling and risk factors assessment of cutaneous leishmaniasis in an expanding endemic focus of Punjab, Pakistan. *Parasitology Research* 124(11), 126. 10.1007/s00436-025-08581-2 41205103 PMC12596330

[ref16] Atnafu A, Chanyalew Z, Yimam S, Zeleke M, Negussie S, Girma S, Melaku A and Chanyalew M (2024) Histopathological patterns of cutaneous and mucocutaneous Leishmaniasis due to L. aethiopica. *Dermatology Research and Practice* 2024(1), 5267606. 10.1155/drp/5267606 39650143 PMC11623993

[ref17] Auwera G VD and Dujardin J-C (2015) Species Typing in Dermal Leishmaniasis. *Clinical Microbiology Reviews* 28(2), 265–294. 10.1128/CMR.00104-14 25672782 PMC4402951

[ref18] Avni D, Solomon M, Strauss M, Sagi O, Temper V, Michael-Gayego A, Meningher T, Avitan-Hersh E, Szwarcwort-Cohen M, Moran-Gilad J, Ollech A and Schwartz E (2024) The epidemiology of PCR-confirmed cutaneous Leishmaniasis in Israel: a nationwide study. *Microorganisms* 12(10), 1950. 10.3390/microorganisms12101950 39458260 PMC11509168

[ref19] Azam M, Singh S, Gupta R, Mayank M, Kathuria S, Sharma S, Ramesh V and Singh R (2024) Assessment of high-resolution melting curve analysis for Leishmania spp. detection in different clinical manifestations of Leishmaniasis in India. *Pathogens* 13(9), 759. 10.3390/pathogens13090759 39338950 PMC11435223

[ref20] Azami-Conesa I, Matas Méndez P, Pérez-Moreno P, Carrión J, Alunda JM, Mateo Barrientos M and Gómez-Muñoz MT (2024) Wildlife as a sentinel for pathogen introduction in nonendemic areas: first detection of Leishmania tropica in Wildlife in Spain. *Transboundary and Emerging Diseases* 2024, 8259712. 10.1155/2024/8259712 40303102 PMC12017196

[ref21] Bachmann I, Behrmann O, Klingenberg-Ernst M, Rupnik M, Hufert FT, Dame G and Weidmann M (2024) Rapid isothermal detection of pathogenic clostridioides difficile using recombinase polymerase amplification. *Analytical Chemistry* 96(8), 3267–3275. 10.1021/acs.analchem.3c02985 38358754

[ref22] Baghad B, Mouhsine Z, Chekairi FZ, Amine B, Saik EII, Lemkhayar K, Lemrani M, Soussi Abdallaoui M, Chiheb S and Riyad M (2025) New epidemio-clinical insights into cutaneous leishmaniasis caused by Leishmania infantum in Casablanca, Morocco. *Infectious Diseases Now* 55(6), 105111. 10.1016/j.idnow.2025.105111 40615082

[ref23] Bamorovat M, Sharifi I, Tahmouresi A, Agha Kuchak Afshari S and Rashedi E (2025) Unlocking responsive and unresponsive signatures: a transfer learning approach for automated classification in cutaneous Leishmaniasis lesions. *Transboundary and Emerging Diseases* 2025(1), 5018632. 10.1155/tbed/5018632 40302757 PMC12016710

[ref24] Bel Hadj Ali I, Saadi-Ben Aoun Y, Hammami Z, Rhouma O, Chakroun AS and Guizani I (2023) Handheld ultra-fast duplex polymerase chain reaction assays and lateral flow detection and identification of leishmania parasites for cutaneous leishmaniases diagnosis. *Pathogens* 12(11), 1292. 10.3390/pathogens12111292 38003756 PMC10675497

[ref25] Bel Hadj Ali I, Saadi-Ben Aoun Y, Khammeri I, Souguir H, Harigua-Souiai E, Chouaieb H, Chakroun AS, Lemrani M, Kallel A, Kallel K, Haddad N, El Dbouni O, Coler RN, Reed SG, Fathallah-Mili A and Guizani I (2024) Recombinase-based amplification coupled with lateral flow chromatography for the specific and sensitive detection and identification of Leishmania major in cutaneous leishmaniasis patients. *Frontiers in Microbiology* 15, 1514684. 10.3389/fmicb.2024.1514684 39931278 PMC11807989

[ref26] Bhattarai NR, Rai K, Uranw S, Khadka DK, Khanal B, Dahal G, Pradhan S, Dhakal S, Monsieurs P, de Gooyer T, Cloots K, Hasker E and Van der Auwera G (2025) Can cutaneous Leishmaniasis provoke a resurgence of kala-azar in the Indian subcontinent? *BMC Infectious Diseases* 25(1), 281. 10.1186/s12879-025-10664-5 40012028 PMC11866896

[ref27] Birtek MT, Atçeken N and Tasoglu S (2025) Programmable 3DP microfluidic bio-reaction system: automated LAMP-on-a-Chip. *Lab on a Chip* 25(21), 5506–23. 10.1039/d5lc00003c. 40779684

[ref28] Blaizot R, Lamine MM, Saout M, Issa K, Laminou IM, Duvignaud A, Demar M and Doutchi M (2025) Molecular detection of Leishmania major human infections in the Zinder area, Niger. *BMC Infectious Diseases* 25(1), 871. 10.1186/s12879-025-11229-2 40597737 PMC12219134

[ref29] Blaizot R, Pasquier G, Kone AK, Duvignaud A and Demar M (2024) Cutaneous leishmaniasis in sub-Saharan Africa: A systematic review of Leishmania species, vectors and reservoirs. *Parasites and Vectors* 17(1), 318. 10.1186/s13071-024-06381-8 39044228 PMC11267819

[ref30] Botha JC, Zafilaza K, Soulie C, Yin N, Spyer M, Balaska S, Chatziioannidou S, Tsiakalou V, Papadakis G, Skoura L, Zafiropoulos A, Sourvinos G, Vandenberg O, Marcelin A-G, Gizeli E and Nastouli E (2024) Evaluation of a near-patient SARS-CoV-2 novel rapid diagnostic platform. *Microbiology Spectrum* 12(12), e00672–24. 10.1128/spectrum.00672-24 39422469 PMC11619423

[ref31] Boulal B, Remadi L, Grigoraki L, Vontas J and Bendjoudi D (2025) Cutaneous Leishmaniasis in southeastern algeria: clinical, parasitological diagnosis and molecular identification. *Acta Parasitologica* 70(6), 234. 10.1007/s11686-025-01178-w 41264036

[ref32] Bruno F, Castelli G, Li B, Reale S, Carra E, Vitale F, Scibetta S, Calzolari M, Varani S, Ortalli M, Franceschini E, Gennari W, Rugna G and Späth GF (2024) Genomic and epidemiological evidence for the emergence of a L. infantum/L. donovani hybrid with unusual epidemiology in northern Italy. *MBio* 15(7), e00995–24. 10.1128/mbio.00995-24 38832792 PMC11253594

[ref33] Bussotti G, Gouzelou E, Boité MC, Kherachi I, Harrat Z, Eddaikra N, Mottram JC, Antoniou M, Christodoulou V, Bali A, Guerfali FZ, Laouini D, Mukhtar M, Dumetz F, Dujardin JC, Smirlis D, Lechat P, Pescher P, Hamouchi AE, Lemrani M, Chicharro C, Llanes-Acevedo IP, Botana L, Cruz I, Moreno J, Jeddi F, Aoun K, Bouratbine A, Cupolillo E and Späth GF (2018) Leishmania genome dynamics during environmental adaptation reveal strain-specific differences in gene copy number variation, karyotype instability, and telomeric amplification. *MBio* 9(6), 10–128. 10.1128/mBio.01399-18 PMC6222132 30401775

[ref34] Camargo BD, Cassaboni Stracke M, Soligo Sanchuki HB, de Oliveira VK, Ancelmo HC, Mozaner Bordin D, Klerynton Marchini F, Ribeiro Viana E and Blanes L (2024) Low-cost arduino reverse transcriptase loop-mediated isothermal amplification (RT-LAMP) for sensitive nucleic acid detection. *Biosensors* 14(3), 128. 10.3390/bios14030128 38534235 PMC10968452

[ref35] Castellanos LR, Chaffee R, Kumar H, Mezgebo BK, Kassau P, Peirano G, Pitout JDD, Kim K and Pillai DR (2024) A novel machine-learning aided platform for rapid detection of urine ESBLs and carbapenemases: URECA-LAMP. *Journal of Clinical Microbiology* 62(11), e0086924. 10.1128/jcm.00869-24 39445836 PMC11559160

[ref36] Castellanos-Gonzalez A, Cossio A, Jojoa J, Moen S and Travi BL (2023) MiniPCR as a portable equipment for the molecular diagnosis of American cutaneous leishmaniasis. *Acta Tropica* 243, 106926. 10.1016/j.actatropica.2023.106926 37088354

[ref37] Cavuto ML, Malpartida-Cardenas K, Pennisi I, Pond MJ, Mirza S, Moser N, Comer M, Stokes I, Eke L, Lant S, Szostak-Lipowicz KM, Miglietta L, Stringer OW, Mantikas KT, Sumner RP, Bolt F, Sriskandan S, Holmes A, Georgiou P, Ulaeto DO, Maluquer de Motes C and Rodriguez-Manzano J (2025) Portable molecular diagnostic platform for rapid point-of-care detection of mpox and other diseases. *Nature Communications* 16(1), 1–3. 10.1038/s41467-025-57647-3 PMC11933461 40128193

[ref38] Chaouch M, Aoun K, Othman SB, Abid MB, Sghaier IB, Bouratbine A and Abderrazak SB (2019) Development and assessment of leishmania major and leishmania tropica specific loop-mediated isothermal amplification assays for the diagnosis of cutaneous leishmaniasis in Tunisia. *American Journal of Tropical Medicine and Hygiene* 101(1), 101–107. 10.4269/ajtmh.19-0097 31094311 PMC6609195

[ref39] Chaouch M, Fathallah-Mili A, Driss M, Lahmadi R, Ayari C, Guizani I, Ben Said M and BenAbderrazak S (2013) Identification of Tunisian Leishmania spp. by PCR amplification of cysteine proteinase B (cpb) genes and phylogenetic analysis. *Acta Tropica* 125(3), 357–365. 10.1016/j.actatropica.2012.11.012 23228525

[ref40] Contreras-Ramírez M, Sora-Cardenas J, Colorado-Salamanca C, Ovalle-Bracho C and Suárez DR (2024) Enhanced detection of leishmania parasites in microscopic images using machine learning models. *Sensors* 24(24), 8180. 10.3390/s24248180 39771915 PMC11679136

[ref41] Cossio A, Bautista-Gomez MM, Alexander N, Del Castillo AM, Del Mar Castro M, Castaño-Grajales PY, Gutiérrez-Poloche YH, Zuluaga LS, Vargas-Bernal L, Navarro A and Saravia NG (2023) mHealth monitoring of treatment of cutaneous Leishmaniasis patients: a community-based implementation study. *American Journal of Tropical Medicine and Hygiene* 109(4), 778–790. 10.4269/ajtmh.22-0805 37640290 PMC10551068

[ref42] Cossio A, Jojoa J, Castro MDM, Castillo RM, Osorio L, Shelite TR, Saravia NG, Melby PC and Travi BL (2021) Diagnostic performance of a recombinant polymerase amplification test—lateral flow (Rpa-lf) for cutaneous leishmaniasis in an endemic setting of Colombia. *PLoS Neglected Tropical Diseases* 15(4), e0009291. 10.1371/journal.pntd.0009291 33909619 PMC8081229

[ref43] Crego-Vicente B, Fernández-Soto P, García-Bernalt Diego J, Febrer-Sendra B and Muro A (2023) Development of a duplex LAMP assay with probe-based readout for simultaneous real-time detection of schistosoma mansoni and Strongyloides spp. -A laboratory approach to point-of-care. *International Journal of Molecular Sciences* 24(1), 893. 10.3390/ijms24010893 36614336 PMC9821331

[ref44] Cruz I, Albertini A, Barbeitas M, Arana B, Picado A, Ruiz-Postigo JA and Ndung’u JM (2019) Target Product Profile for a point-of-care diagnostic test for dermal leishmaniases. *Parasite Epidemiology and Control* 5, e00103. 10.1016/j.parepi.2019.e00103 30923755 PMC6423987

[ref45] Cruz-Saavedra L, Ospina C, Patiño LH, Villar JC, Sáenz Pérez LD, Cantillo-Barraza O, Jaimes-Dueñez J, Ballesteros N, Cáceres T, Vallejo G and Ramírez JD (2024) Enhancing trypanosomatid identification and genotyping with oxford nanopore sequencing: development and validation of an 18S rRNA amplicon-based method. *Journal of Molecular Diagnostics* 26(5), 323–336. 10.1016/j.jmoldx.2024.01.012 38360211

[ref46] Cui J, Tian A, Wang H, Yu Y, Hao J, Wang L, Shi C and Ma C (2025a) Hydrogel loop-mediated isothermal amplification for ultra-fast diagnosis of Helicobacter pylori in stool samples without nucleic acid extraction. *Analytica Chimica Acta* 1333, 343384. 10.1016/j.aca.2024.343384 39615902

[ref47] Cui S, Wang K, Yang Y, Lv X and Li X (2025b) An integrated and paper-based microfluidic system employing LAMP-CRISPR and equipped with a portable device for simultaneous detection of pathogens. *Analytical and Bioanalytical Chemistry* 417(4), 785–797. 10.1007/s00216-024-05693-z 39710780

[ref48] Dai F, Zhang T, Pang F, Jiao T, Wang K, Zhang Z, Wang N, Xie Z, Zhang Y, Wang Z, Chen Z, Yu M, Wei H and Song J (2025) A compact, palm-sized isothermal fluorescent diagnostic intelligent IoT device for personal health monitoring and beyond via one-tube/one-step LAMP-CRISPR assay. *Biosensors and Bioelectronics* 270, 116945. 10.1016/j.bios.2024.116945 39577180

[ref49] Danthanarayana AN, Nandy S, Kourentzi K, Vu B, Shelite TR, Travi BL, Brgoch J and Willson RC (2023) Smartphone-readable RPA-LFA for the highsensitivity detection of Leishmania kDNA using nanophosphor reporters. *PLoS Neglected Tropical Diseases* 17(7), e0011436. 10.1371/journal.pntd.0011436 37399214 PMC10353800

[ref50] Das D, Lin CW and Chuang HS (2022) LAMP-Based Point-of-Care Biosensors for Rapid Pathogen Detection. *Biosensors* 12(12), 1068. 10.3390/bios12121068 36551035 PMC9775414

[ref51] de Almeida JV, de Souza CF, Fuzari AA, Joya CA, Valdivia HO, Bartholomeu DC and Brazil RP (2021) Diagnosis and identification of Leishmania species in patients with cutaneous leishmaniasis in the state of Roraima, Brazil’s Amazon Region. *Parasites and Vectors* 14(1), 32. 10.1186/s13071-020-04539-8 33413563 PMC7791761

[ref52] de Avelar DM, Santos CC and Fusaro Faioli A (2023) Developments in Leishmaniasis diagnosis: A patent landscape from 2010 to 2022. *PLOS Global Public Health* 3(11), e0002557. 10.1371/journal.pgph.0002557 37910459 PMC10619796

[ref53] de Lima LF, Estrela PFN, Faustino LC, Souza JV, Justino de Assunção C, Gerôncio ETS, Fonseca de Lima L, Luiz Cardoso Bailão EF, de Araujo WR, Paixão TRLC and Duarte GRM (2025) 3D-printed electrochemical loop-mediated isothermal amplification (E-LAMP): A miniaturized and portable platform to detect methicillin-resistant Staphylococcus aureus (MRSA). *Analytica Chimica Acta* 1372, 1–9. 10.1016/j.aca.2025.344455 40903125

[ref54] De Los Santos MB, Loyola S, Perez-Velez ES, Santos RDP, Ramírez IM and Valdivia HO (2024) Sampling is decisive to determination of Leishmania (Viannia) species. *PLoS Neglected Tropical Diseases* 18(4), e0012113. 10.1371/journal.pntd.0012113 38662642 PMC11045131

[ref55] de Vries HJC and Schallig HD (2022) Cutaneous Leishmaniasis: a 2022 updated narrative review into diagnosis and management developments. *American Journal of Clinical Dermatology. Adis* 23(6), 823–840. 10.1007/s40257-022-00726-8 PMC9472198 36103050

[ref56] Dey R, Alshaweesh J, Singh KP, Lypaczewski P, Karmakar S, Klenow L, Paulini K, Kaviraj S, Kamhawi S, Valenzuela JG, Singh S, Hamano S, Satoskar AR, Gannavaram S, Nakhasi HL and Matlashewski G (2023) Production of leishmanin skin test antigen from Leishmania donovani for future reintroduction in the field. *Nature Communications* 14(1), 7028. 10.1038/s41467-023-42732-2 PMC10622560 37919280

[ref57] Di Altobrando A, Misciali C, Raone B, Attard L and Gaspari V (2021) Case report: Cutaneous leishmaniasis misdiagnosed as pyoderma gangrenosum. *American Journal of Tropical Medicine and Hygiene* 104(2), 640–642. 10.4269/ajtmh.20-0735 PMC7866308 33319736

[ref58] Donadeu M, Gyorkos TW, Horstick O, Lammie P, Mwingira UJ, Omondi WP, Pritchard JM, Tappero J, Chan YYL, Welsche S, Agua JFV, Madjou S, Mbabazi PS, Warusavithana S and Torres-Vitolas CA (2025) Tracking progress along the WHO Neglected tropical diseases road map to 2030: A guide to the Gap Assessment Tool (GAT) and results from the 2023-2024 assessment. *PLoS Neglected Tropical Diseases* 19(7), e0013194. 10.1371/journal.pntd.0013194 40591727 PMC12244624

[ref59] Dong H, Mo J, Yu Y, Xie W, Zheng J and Jia C (2023) A portable system for economical nucleic acid amplification testing. *Frontiers in Bioengineering and Biotechnology* 11, 1214624. 10.3389/fbioe.2023.1214624 37600301 PMC10436208

[ref60] Dong M, Kshirsagar A, Politza AJ and Guan W (2024a) High fidelity machine-learning-assisted false positive discrimination in loop-mediated isothermal amplification using nanopore-based sizing and counting. *ACS Nano* 18(9), 7170–7179. 10.1021/acsnano.3c12053 38393338 PMC11197460

[ref61] Dong T, Qin L, Wang Z, Fan C, Shen C, Feng P, Kong Q, Ke B, Ying B and Li F (2024b) Point-of-care diagnosis of tuberculosis using a portable nucleic acid test with distance-based readout. *Analytical Chemistry* 96(51), 20204–20212. 10.1021/acs.analchem.4c04180 39665389

[ref62] Dueñas E, Nakamoto JA, Cabrera-Sosa L, Huaihua P, Cruz M, Arévalo J, Milón P and Adaui V (2022) Novel CRISPR-based detection of Leishmania species. *Frontiers in Microbiology* 13, 958693. 10.3389/fmicb.2022.958693 36187950 PMC9520526

[ref63] EL Banna G, Braun N, Baker NM, Peacker BL, Ma KSK, Kibbi AG and Chen ST (2025) The burden of skin and subcutaneous diseases in North Africa and the Middle East (NAME) from 1990 to 2021. *International Journal of Dermatology* 64(12):2274–82. 10.1111/ijd.17953 40619585 PMC12483311

[ref64] El Moctar A, Ouldabdallahi Moukah M, Koné AK, Saout M, Demar M, Fofana MD, Cheikh Mohamed Vadel TK, Kébé M, Thera MA, Blaizot R and Ould Mohamed Salem Boukhary A (2025) Ongoing presence of Leishmania major cutaneous leishmaniasis in Mauritania, 2016–2024. *BMC Infectious Diseases* 25(1), 596. 10.1186/s12879-025-10716-w 40275194 PMC12023448

[ref65] Feddema JJ, Fernald KDS, Keijser BJF, Kieboom J and van de Burgwal LHM (2024) Commercial opportunity or addressing unmet needs—Loop-Mediated Isothermal Amplification (LAMP) as the future of rapid diagnostic testing? *Diagnostics* 14(17), 1845. 10.3390/diagnostics14171845 39272630 PMC11394392

[ref66] Fowler VL, Armson B, Gonzales JL, Wise EL, Howson ELA, Vincent-Mistiaen Z, Fouch S, Maltby CJ, Grippon S, Munro S, Jones L, Holmes T, Tillyer C, Elwell J, Sowood A, de Peyer O, Dixon S, Hatcher T, Patrick H, Laxman S, Walsh C, Andreou M, Morant N, Clark D, Moore N, Houghton R, Cortes NJ and Kidd SP (2021) A highly effective reverse-transcription loop-mediated isothermal amplification (RT-LAMP) assay for the rapid detection of SARS-CoV-2 infection. *Journal of Infection* 82(1), 117–125. 10.1016/j.jinf.2020.10.039 33271166 PMC7703389

[ref67] Freire ML, Machado de Assis T, Oliveira E, Moreira de Avelar D, Siqueira IC, Barral A, Rabello A and Cota G (2019) Performance of serological tests available in Brazil for the diagnosis of human visceral leishmaniasis. *PLoS Neglected Tropical Diseases* 13(7), e0007484. 10.1371/journal.pntd.0007484 31318856 PMC6638734

[ref68] Frej A, Cano M, Ruiz-Postigo JA, Macharia P, Phillips RO, Amoako YA and Carrion C (2022) Assessing the quality of the world health organization’s skin NTDs App as a training tool in Ghana and Kenya: Protocol for a cross-sectional study. *JMIR Research Protocols* 11(12), e39393. 10.2196/39393 36480252 PMC9782345

[ref69] Gadri S, Bounab S, Benazi N and Zerouak F (2025) A new diagnostic method and tool for cutaneous leishmaniasis based on artificial intelligence techniques. *Computers in Biology and Medicine* 192, 110313. 10.1016/j.compbiomed.2025.110313 40359677

[ref70] Ge H, Feng J, Huang L, Luo Z, Ling H, Ma L, Wang M, Chen H and Ren L (2025) Development of a highly sensitive, high-throughput and automated CRISPR-based device for the contamination-free pathogen detection. *Biosensors and Bioelectronics* 278, 117323. 10.1016/j.bios.2025.117323 40055023

[ref71] Gebremeskele BT, Adane G, Adem M and Tajebe F (2023) Diagnostic performance of CL Detect rapid-immunochromatographic test for cutaneous leishmaniasis: A systematic review and meta-analysis. *Systematic Reviews* 12(1), 240. 10.1186/s13643-023-02422-y 38115138 PMC10731771

[ref72] Georgiadou SP, Makaritsis KP and Dalekos GN (2015) Leishmaniasis revisited: current aspects on epidemiology, diagnosis and treatment. *Journal of Translational Internal Medicine* 3(2), 43–50. 10.1515/jtim-2015-0002 27847886 PMC4936444

[ref73] Ghosh P, Chowdhury R, Faisal K, Khan MAA, Hossain F, Rahat MA, Chowdhury MAA, Mithila NT, Kamal M, Maruf S, Nath R, Kobialka RM, Ceruti A, Cameron M, Duthie MS, Wahed AAE and Mondal D (2023) Evaluation of a point-of-need molecular diagnostic tool coupled with rapid DNA extraction methods for Visceral Leishmaniasis. *Diagnostics* 13(24), 3639. 10.3390/diagnostics13243639 38132223 PMC10742398

[ref74] Gonzalez-Garcia LN, Rodríguez-Guzmán AM, Vargas-León CM, Aponte S, Bonilla-Valbuena LA, Matiz-González JM, Clavijo-Vanegas AM, Duarte-Olaya GA, Aguilar-Buitrago C, Urrea DA, Duitama J and Echeverry MC (2025) Genomic characterization of Leishmania (V.) braziliensis associated with antimony therapeutic failure and variable in vitro tolerance to amphotericin B. *Scientific Reports* 15(1), 12973. 10.1038/s41598-025-96849-z 40234696 PMC12000620

[ref75] Grogl M, Joya CA, Saenz M, Quispe A, Rosales LA, Santos RDP, De Los Santos MB, Donovan N, Ransom JH, Ramos A and Cuentas EL (2023) Evaluation of a diagnostic device, CL Detect rapid test for the diagnosis of new world cutaneous leishmaniasis in Peru. *PLoS Neglected Tropical Diseases* 17(3), e0011054. 10.1371/journal.pntd.0011054 36913433 PMC10010545

[ref76] Gurel MS, Tekin B and Uzun S (2020) Cutaneous leishmaniasis: A great imitator. *Clinics in Dermatology* 38(2), 140–151. 10.1016/j.clindermatol.2019.10.008 32513395

[ref77] Hadermann A, Heeren S, Maes I, Dujardin JC, Domagalska MA and Van den Broeck F (2023) Genome diversity of Leishmania aethiopica. *Frontiers in Cellular & Infection Microbiology* 13, 1147998. 10.3389/fcimb.2023.1147998 37153154 PMC10157169

[ref78] Hagos DG, Kiros YK, Abdulkader M, Arefaine ZG, Nigus E, Schallig HHDF and Wolday D (2021) Utility of the loop-mediated isothermal amplification assay for the diagnosis of visceral leishmaniasis from blood samples in Ethiopia. *American Journal of Tropical Medicine and Hygiene* 105(4), 1050–1055. 10.4269/ajtmh.21-0334 34310340 PMC8592144

[ref79] Hagos DG, Kiros YK, Abdulkader M, Schallig HDFH and Wolday D (2024) Comparison of the diagnostic performances of five different tests in diagnosing Visceral Leishmaniasis in an endemic region of Ethiopia. *Diagnostics* 14(2), 163. 10.3390/diagnostics14020163 38248040 PMC10813839

[ref80] Harigua-Souiai E, Salem YB, Hariga M, Saadi Y, Souguir H, Chouaieb H, Adedokun O, Mkada I, Moussa Z, Fathallah-Mili A, Lemrani M, Haddad N, Oduola A, Souiai O, Ali IBH and Guizani I (2025) Lesionia: a digital data management system to enhance collaborative management of epidemiological and clinical data of cutaneous leishmaniases patients. *BMC Research Notes* 18(1), 160. 10.1186/s13104-025-07208-0 40217269 PMC11987383

[ref81] Hayatolgheib-Moghadam S, Pourzandkhanooki M, Hadighi R, Geraili A, Alipour M, Namrodi J, Rampisheh Z and Badirzadeh A (2025) Epidemiological investigation and diagnostic comparison of cutaneous leishmaniasis in Kalaleh city, Iran: a parasitological and molecular approach. *Journal of Parasitic Diseases* 49(2), 351–358. 10.1007/s12639-024-01753-4 40458495 PMC12126409

[ref82] Heiniger EK, Jiang KP, Kumar S and Yager P (2025) A low-cost point-of-care device for the simultaneous detection of two sexually transmitted bacterial pathogens in vaginal swab samples. *The Analyst* 150(19), 4414–4426. 10.1039/D5AN00496A 40910374 PMC12412115

[ref83] Hietanen H, Pfavayi LT and Mutapi F (2025) Unlocking the blueprint to eliminating neglected tropical diseases: a review of efforts in 50 countries that have eliminated at least 1 NTD. *PLoS Neglected Tropical Diseases* 19(9):e0013424. 10.1371/journal.pntd.0013424 40906712 PMC12410759

[ref84] Ho M, Sathishkumar N, Sklavounos AA, Sun J, Yang I, Nichols KP and Wheeler AR (2023) Digital microfluidics with distance-based detection - a new approach for nucleic acid diagnostics. *Lab on a Chip* 24(1), 63–73. 10.1039/d3lc00683b 37987330

[ref85] Hossain F, Picado A, Owen SI, Ghosh P, Chowdhury R, Maruf S, Khan MAA, Rashid MU, Nath R, Baker J, Ghosh D, Adams ER, Duthie MS, Hossain MS, Basher A, Nath P, Aktar F, Cruz I and Mondal D (2021) Evaluation of Loopamp^TM^ Leishmania detection kit and Leishmania Antigen ELISA for post-elimination detection and management of Visceral Leishmaniasis in Bangladesh. *Frontiers in Cellular & Infection Microbiology* 11, 670759. 10.3389/fcimb.2021.670759 33981632 PMC8108992

[ref86] Hosseini-Safa A, Mohebali M, Hajjaran H, Akhoundi B, Zarei Z, Arzamani K and Davari A (2018) High resolution melting analysis as an accurate method for identifying Leishmania infantum in canine serum samples. *Journal of Vector Borne Diseases* 55(4), 315. 10.4103/0972-9062.256568 30997893

[ref87] Hoyos J, Rosales-Chilama M, León C, González C and Gómez MA (2022) Sequencing of hsp70 for discernment of species from the Leishmania (Viannia) guyanensis complex from endemic areas in Colombia. *Parasites and Vectors* 15(1), 406. 10.1186/s13071-022-05438-w 36329517 PMC9635106

[ref88] Hu F, Zhang Y, Yang Y, Peng L, Cui S, Ma Q, Wang F and Wang X (2025) A rapid and ultrasensitive RPA-assisted CRISPR–Cas12a/Cas13a nucleic acid diagnostic platform with a smartphone-based portable device. *Biosensors and Bioelectronics* 280, 117428. 10.1016/j.bios.2025.117428 40179699

[ref89] Huggins LG, Colella V, Young ND and Traub RJ (2024) Metabarcoding using nanopore long-read sequencing for the unbiased characterization of apicomplexan haemoparasites. *Molecular Ecology Resources* 24(2), e13878. 10.1111/1755-0998.13878 37837372

[ref90] Iantorno SA, Durrant C, Khan A, Sanders MJ, Beverley SM, Warren WC, Berriman M, Sacks DL, Cotton JA and Grigg ME (2017) Gene expression in Leishmania is regulated predominantly by gene dosage. *MBio* 8(5), 10–128. 10.1128/mBio.01393-17 PMC5596349 28900023

[ref91] Iatta R, Mendoza-Roldan JA, Latrofa MS, Cascio A, Brianti E, Pombi M, Gabrielli S and Otranto D (2021) Leishmania tarentolae and leishmania infantum in humans, dogs and cats in the pelagie archipelago, southern Italy. *PLoS Neglected Tropical Diseases* 15(9), e0009817. 10.1371/journal.pntd.0009817 34555036 PMC8491888

[ref92] Ibarra-Meneses AV, Cruz I, Chicharro C, Sánchez C, Biéler S, Broger T, Moreno J and Carrillo E (2018) Evaluation of fluorimetry and direct visualization to interpret results of a loop-mediated isothermal amplification kit to detect Leishmania DNA. *Parasites and Vectors* 11(1), 250. 10.1186/s13071-018-2836-2 29665825 PMC5905109

[ref93] Imai K, Tarumoto N, Amo K, Takahashi M, Sakamoto N, Kosaka A, Kato Y, Mikita K, Sakai J, Murakami T, Suzuki Y, Maesaki S and Maeda T (2018) Non-invasive diagnosis of cutaneous leishmaniasis by the direct boil loop-mediated isothermal amplification method and MinION^TM^ nanopore sequencing. *Parasitology International* 67(1), 34–37. 10.1016/j.parint.2017.03.001 28288843

[ref94] Jaimes J, Patiño LH, Herrera G, Cruz C, Pérez J, Correa-Cárdenas CA, Muñoz M and Ramírez JD (2024) Prokaryotic and eukaryotic skin microbiota modifications triggered by Leishmania infection in localized Cutaneous Leishmaniasis. *PLoS Neglected Tropical Diseases* 18(3), e0012029. 10.1371/journal.pntd.0012029 38478569 PMC10962849

[ref95] Jain M, Sangma DA, Parida L, Negi R, Negi A, Matlashewski G and Lypaczewski P (2024a) Atypical cutaneous leishmaniasis: a new challenge to VL elimination in South-East Asia. *Frontiers in Cellular & Infection Microbiology* 14, 1454002. 10.3389/fcimb.2024.1454002 39559700 PMC11570545

[ref96] Jain S, Madjou S and Agua JFV (2024b) Global leishmaniasis surveillance updates 2023: 3 years of the NTD road map. *Weekly Epidemiological Record* 17. https://iris.paho.org/ (accessed 10 June 2025)

[ref97] Jiang H, Zhu X, Jiao J, Yan C, Liu K, Chen W and Qin P (2025) CRISPR/dCas9-based hotspot self-assembling SERS biosensor integrated with a smartphone for simultaneous, ultrasensitive and robust detection of multiple pathogens. *Biosensors and Bioelectronics* 270, 116974. 10.1016/j.bios.2024.116974 39586143

[ref98] Jin M, Ding J, Zhou Y, Chen J, Wang Y and Li Z (2024) StratoLAMP: label-free, multiplex digital loop-mediated isothermal amplification based on visual stratification of precipitate. *Proceedings of the National Academy of Sciences of the United States of America* 121(2), e2314030121. 10.1073/pnas.2314030121 38165933 PMC10786297

[ref99] Jin N, Yang F, Zhang X, Li Y and Lin J (2025) Sensitive detection of multiplex bacteria based on finger driven microfluidics and recombinase aided amplification. *Biosensors and Bioelectronics* 287, 117750. 10.1016/j.bios.2025.117750 40639142

[ref100] Kämink S, Masih B, Ali N, Ullah A, Khan SJ, Ashraf S, Pylypenko T, Grobusch MP, Fernhout J, Boer MD and Ritmeijer K (2021) Effectiveness of miltefosine in cutaneous leishmaniasis caused by leishmania tropica in Pakistan after antimonial treatment failure or contraindications to first line therapy—a retrospective analysis. *PLoS Neglected Tropical Diseases* 15(1), 1–14. 10.1371/journal.pntd.0008988 PMC7872246 33507944

[ref101] Karaja S, Halloum M, Karaja S, Daher Alhussen A, hadiQatza A, Mansour S and Almasri AA (2024) Cutaneous leishmaniasis mimicking psoriasis: a case report. *Clinical Case Reports* 12(8), e9299. 10.1002/ccr3.9299 39119034 PMC11305983

[ref102] Khedhiri M, Chaouch M, Ayouni K, Chouikha A, Gdoura M, Touzi H, Hogga N, Benkahla A, Fares W and Triki H (2024) Development and evaluation of an easy to use real-time reverse-transcription loop-mediated isothermal amplification assay for clinical diagnosis of West Nile virus. *Journal of Clinical Virology* 170, 105633. 10.1016/j.jcv.2023.105633 38103483

[ref103] Kim HE, Schuck A, Park H, Chung DR, Kang M and Kim YS (2024) Dual-mode graphene field-effect transistor biosensor with isothermal nucleic acid amplification. *Biosensors* 14(2), 91. 10.3390/bios14020091 38392010 PMC10886465

[ref104] Kim J, Zieneldien T, Ma S and Cohen BA (2025) Cutaneous Leishmaniasis in the context of global travel, migration, refugee populations, and humanitarian crises. *Clinics and Practice* 15(4), 77. 10.3390/clinpract15040077 40310302 PMC12025697

[ref105] Ko MY, Fowler E, Scott A and Uslan DZ (2025) Miltefosine failure and amphotericin B success in the treatment of a case of cutaneous Leishmania Braziliensis in a recent traveler in Belize and Guatemala. *Case Reports in Infectious Diseases* 2025(1), 6644758. 10.1155/crdi/6644758 40656817 PMC12255491

[ref106] Kobialka RM, Ceruti A, Roy M, Roy S, Chowdhury R, Ghosh P, Hossain F, Weidmann M, Graf E, Bueno Alvarez J, Moreno J, Truyen U, Mondal D, Chatterjee M and Abd El Wahed A (2024) Portable smartphone-based molecular test for rapid detection of Leishmania spp. *Infection* 52(4), 1315–1324. 10.1007/s15010-024-02179-z 38353873 PMC11288998

[ref107] Koutsogiannis Z and Denny PW (2024) Rapid genotyping of Toxoplasma gondii isolates via Nanopore-based multi-locus sequencing. *AMB Express* 14(1), 68. 10.1186/s13568-024-01728-x 38844693 PMC11156620

[ref108] Krayter L, Bumb RA, Azmi K, Wuttke J, Malik MD, Schnur LF, Salotra P and Schönian G (2014) Multilocus microsatellite typing reveals a genetic relationship but, also, genetic differences between Indian strains of Leishmania tropica causing cutaneous leishmaniasis and those causing visceral leishmaniasis. *Parasites and Vectors* 7(1), 123. 10.1186/1756-3305-7-123 24666968 PMC3987047

[ref109] Krayter L, Schnur LF and Schönian G (2015) The genetic relationship between leishmania aethiopica and leishmania tropica revealed by comparing microsatellite profiles. *PLoS ONE* 10(7), e0131227. 10.1371/journal.pone.0131227 26196393 PMC4511230

[ref110] Kshirsagar A, DeRosa D, Politza AJ, Liu T, Dong M and Guan W (2025) Point-of-need one-pot multiplexed RT-LAMP test for detecting three common respiratory viruses in saliva. *Biosensors and Bioelectronics* 288, 1–13. 10.1016/j.bios.2025.117836 PMC12395363 40768948

[ref111] Kumar N, Kumari M, Chander D, Dogra S, Chaubey A, Chakraborty S and Arun RK (2025) Portable, quantitative, real-time isothermal nucleic acid amplification test using microfluidic device-coupled UV-LED photodiode detector. *Biosensors and Bioelectronics* 274, 117194. 10.1016/j.bios.2025.117194 39904093

[ref112] Kumar Y, Garg P, Moudgil MR, Singh R, Woźniak M, Shafi J and Ijaz MF (2024) Enhancing parasitic organism detection in microscopy images through deep learning and fine-tuned optimizer. *Scientific Reports* 14(1), 5753. 10.1038/s41598-024-56323-8 38459096 PMC10923792

[ref113] Kundrod KA, Barra M, Wilkinson A, Smith CA, Natoli ME, Chang MM, Coole JB, Santhanaraj A, Lorenzoni C, Mavume C, Atif H, Montealegre JR, Scheurer ME, Castle PE, Schmeler KM and Richards-Kortum RR (2023) An integrated isothermal nucleic acid amplification test to detect HPV16 and HPV18 DNA in resource-limited settings. *Science Translational Medicine* 15(701), eabn4768. 10.1126/scitranslmed.abn4768 37343083 PMC10566637

[ref114] Leal JFDC, Barroso DH, Trindade NS, Miranda VLD and Gurgel-Gonçalves R (2024) Automated identification of cutaneous leishmaniasis lesions using deep-learning-based artificial intelligence. *Biomedicines* 12(1), 12. 10.3390/biomedicines12010012 PMC10813291 38275373

[ref115] Lee YC, Bu Y, Ni S, Liu Y, Hu A and Yobas L (2025) A compact sample-to-answer system for rapid MRSA detection in serum based on reagent-free electrophoretic purification of nucleic acids and colorimetric LAMP. *Lab on a Chip* 25(19), 5019–5029. 10.1039/D5LC00152H 40827580

[ref116] Lévêque MF, Battery E, Delaunay P, Lmimouni BE, Aoun K, L’ollivier C, Bastien P, Mary C, Pomares C, Fillaux J and Lachaud L (2020) Evaluation of six commercial kits for the serological diagnosis of Mediterranean visceral leishmaniasis. *PLoS Neglected Tropical Diseases* 14(3), e0008139. 10.1371/journal.pntd.0008139 32210438 PMC7135331

[ref117] Li H, Nie Y, Wu Y, Cao Y, Liu W, Zhao R, Feng X and Hao R (2025) Portable microfluidic-LAMP assay for rapid on-site detection of eight highly pathogenic viruses. *Analytica Chimica Acta* 1365, 344236. 10.1016/j.aca.2025.344236 40484541

[ref118] Liang Y, Xie SC, Lv YH, He YH, Zheng XN, Cong W, Elsheikha HM and Zhu XQ (2024) A novel single-tube LAMP-CRISPR/Cas12b method for rapid and visual detection of zoonotic Toxoplasma gondii in the environment. *Infectious Diseases of Poverty* 13(1), 94. 10.1186/s40249-024-01266-5 39654027 PMC11629535

[ref119] Liao Y, Liu Y, Feng Y, Zhen D and He F (2024) Rapid detection of broad-spectrum pathogenic bacteria based on highly sensitive proton response of the nucleic acid amplification SPQC platform. *Analytical Chemistry* 96(17), 6756–6763. 10.1021/acs.analchem.4c00437 38625745

[ref120] Lin L, Xue Y, Tan L, Jiang C, Liu M, Li X, Qiu J, Zhang H, Zhou J and Shu B (2025a) Micro-scale thermofluidics enable autonomous and scalable CRISPR diagnostics for sexually transmitted infections screening. *Biosensors and Bioelectronics* 285, 117591. 10.1016/j.bios.2025.117591 40403612

[ref121] Lin Q, Gou H, Qu X, Jia K, Deng Y, Li D, Cai Q, Liang Y, Xu X, Li Y, Lin J, Li L, Jiang Y, Du S, Deng L, Yan B, Liu R, Li C, Zhang J and Liao M (2025b) A smart single-loop-mediated isothermal amplification facilitates flexible SNP probe design for on-site rapid differentiation of SARS-CoV-2 Omicron variants. *Advanced Science* 12(26), 2502708. 10.1002/advs.202502708 40167300 PMC12245020

[ref122] Lypaczewski P, Thakur L, Jain A, Kumari S, Paulini K, Matlashewski G and Jain M (2022) An intraspecies Leishmania donovani hybrid from the Indian subcontinent is associated with an atypical phenotype of cutaneous disease. *IScience* 25(2), 1–13. 10.1016/j.isci.2022.103802 PMC8841885 35198868

[ref123] Maia de Souza R, Ruedas Martins RC, Moyses Franco LA, Tuon FF, de Oliveira Junior IG, Maia da Silva CA, Imamura R and Amato VS (2022) Identification of Leishmania species by next generation sequencing of hsp70 gene. *Molecular and Cellular Probes* 61, 101791. 10.1016/j.mcp.2022.101791 35051596

[ref124] Malpartida-Cardenas K, Moser N, Ansah F, Pennisi I, Ahu Prah D, Amoah LE, Awandare G, Hafalla JCR, Cunnington A, Baum J, Rodriguez-Manzano J and Georgiou P (2023) Sensitive detection of asymptomatic and symptomatic malaria with seven novel parasite-specific LAMP assays and translation for use at point-of-care. *Microbiology Spectrum* 11(3), e05222–22. 10.1128/spectrum.05222-22 37158750 PMC10269850

[ref125] Mann S, Frasca K, Scherrer S, Henao-Martínez AF, Newman S, Ramanan P and Suarez JA (2021) A review of Leishmaniasis: current knowledge and future directions. *Current Tropical Medicine Reports* 8(2), 121–132. 10.1007/s40475-021-00232-7 33747716 PMC7966913

[ref126] Marvi-Moghadam N, Mohebali M, Rassi Y, Zahraei-Ramazani AR, Oshaghi MA, Jafari R, Fatemi M, Arandian MH, Abdoli H, Shareghi N, Ghanei M, Jalali-Zand N, Veysi A, Ramazanpoor J, Aminian K, Salehi A, Khamesipour A and Akhavan AA (2025) Leishmania spp infection in patients and great gerbils (Rhombomys opimus) in a high-risk focus of zoonotic Cutaneous Leishmaniasis in Central Iran: a microscopic and molecular survey. *Journal of Arthropod-Borne Diseases* 18(3), 253–263. 10.18502/jad.v18i3.18576 PMC12144852 40486262

[ref127] Mata-Somarribas C, Cardoso Das Graças G, de Oliveira Pereira LR, Boité MC, Cantanhêde LM, Braga Filgueira CP, Fallas A, Quirós-Rojas L, Morelli KA, Melim Ferreira GE and Cupolillo E (2024) Applying a cytochrome c oxidase I barcode for Leishmania species typing. *PLoS ONE* 19(12), e0309277. 10.1371/journal.pone.0309277 39621694 PMC11611135

[ref128] Miyajima A, Nishimura F, Natsuhara D, Kiba Y, Okamoto S, Nagai M, Yamamuro T, Kitamura M and Shibata T (2025) Parallel dilution microfluidic device for enabling logarithmic concentration generation in molecular diagnostics. *Lab on a Chip* 25(13), 3242–3253. 10.1039/d5lc00356c 40489078

[ref129] Mizushina H, Imai K, Ohama Y, Sato A, Tanaka M, Omachi R, Takeuchi K, Nakayama SI, Akeda Y and Maeda T (2025) Evaluation of the GeneSoC rapid quantitative PCR system for Treponema pallidum detection. *Journal of Infection and Chemotherapy* 31(8), 102765. 10.1016/j.jiac.2025.102765 40602681

[ref130] Mohamed Noureldeen A, Salem Masoud K and Abulqasim Almakhzoom O (2023) Deep learning model for Cutaneous leishmaniasis detection and classification using Yolov5. *African Journal of Advanced Pure and Applied Sciences (AJAPAS)*. 2(2), 270–280. 10.65418/ajapas.v2i2.382

[ref131] Mohammadi Manesh R, Mousavi S, Mousavi P, Zolfaghari A, Zarei Z, Sharifi I, Zarrinfar H, Hejazi SH, Ataei B, Mohebali M and Mirhendi H (2025) Detection and identification of Leishmania major, Leishmania tropica, and Leishmania infantum in clinical samples based on size polymorphism of partially amplified ribosomal DNA. *The American Journal of Tropical Medicine and Hygiene* 113(6), 1220–1225. 10.4269/ajtmh.24-0843 41056921 PMC12676614

[ref132] Monsieurs P, Cloots K, Uranw S, Banjara MR, Ghimire P, Burza S, Hasker E, Dujardin JC and Domagalska MA (2024) Source tracing of Leishmania donovani in emerging foci of visceral Leishmaniasis, Western Nepal. *Emerging Infectious Diseases* 30(3), 611–613. 10.3201/eid3003.231160 38407178 PMC10902524

[ref133] Moreno-Rodríguez A, Campo-Colín ASMD, Domínguez-Díaz LR, Posadas-Jiménez AL, Matadamas-Martínez F and Yépez-Mulia L (2025) Molecular identification and drug susceptibility of Leishmania spp. clinical isolates collected from two regions of Oaxaca, Mexico. *Microorganisms* 13(2), 220. 10.3390/microorganisms13020220 40005587 PMC11857778

[ref134] Moser N, Yu LS, Rodriguez Manzano J, Malpartida-Cardenas K, Au A, Arkell P, Cicatiello C, Moniri A, Miglietta L, Wang WH, Wang SF, Holmes A, Chen YH and Georgiou P (2022) Quantitative detection of dengue serotypes using a smartphone-connected handheld lab-on-chip platform. *Frontiers in Bioengineering and Biotechnology* 10, 892853. 10.3389/fbioe.2022.892853 36185458 PMC9521504

[ref135] Moungui HC, Tonkoung Iyawa P, Nana-Djeunga H, Ruiz-Postigo JA and Carrion C (2025) Usability and quality evaluation of the World Health Organization SkinNTDs app among frontline health workers in Cameroon: a mixed methods study. *PLoS Neglected Tropical Diseases* 19(9), e0013461. 10.1371/journal.pntd.0013461 40929288 PMC12422481

[ref136] Mughal MAS, Khan MK, Lan H, Abbas RZ, Imran M, Abbas Z, Mehmood MS and Ali S (2025) Decoding Leishmania in equines: a comparative analysis of molecular targets. *Molecular and Biochemical Parasitology* 264, 111699. 10.1016/j.molbiopara.2025.111699 40912367

[ref137] Mukhtar M, Ali SS, Boshara SA, Albertini A, Monnerat S, Bessell P, Mori Y, Kubota Y, Ndung’u JM and Cruz I (2018) Sensitive and less invasive confirmatory diagnosis of visceral leishmaniasis in Sudan using loop-mediated isothermal amplification (LAMP). *PLoS Neglected Tropical Diseases* 12(2), e0006264. 10.1371/journal.pntd.0006264 29444079 PMC5828521

[ref138] Nagamine K, Hase T and Notomi T (2002) Accelerated reaction by loop-mediated isothermal amplification using loop primers. *Molecular and Cellular Probes* 16(3), 223–229. 10.1006/mcpr.2002.0415 12144774

[ref139] Narasimhan V, Kim H, Lee SH, Kang H, Siddique RH, Park H, Wang YM, Choo H, Kim Y and Kumar S (2023) Nucleic acid amplification-based technologies (NAAT)—Toward accessible, autonomous, and mobile diagnostics. *Advanced Materials Technologies* 8(20), 2300230. 10.1002/admt.202300230

[ref140] Nawattanapaibool N, Ruang-Areerate T, Piyaraj P, Leelayoova S, Mungthin M and Siripattanapipong S (2024) Development of nucleic acid lateral flow immunoassay for duplex detection of Leishmania martiniquensis and Leishmania orientalis in asymptomatic patients with HIV. *PLoS ONE* 19(8), e0307601. 10.1371/journal.pone.0307601 39186742 PMC11346928

[ref141] Naz S, Nalcaci M, Hayat O, Toz S, Minhas A, Waseem S and Ozbel Y (2024) Genetic diversity and epidemiological insights into cutaneous leishmaniasis in Pakistan: a comprehensive study on clinical manifestations and molecular characterization of Leishmania species. *Parasitology Research* 123(9), 320. 10.1007/s00436-024-08344-5 39254766

[ref142] Nharwal L, Beg MA, Sehgal D, Singh OP, Tiwari A, Selvapandiyan A and Chouhan G (2025) Emerging trends and Innovative strategies for the diagnosis of Leishmaniasis: a quantum leap from classical to modern era. *Acta Tropica* 270, 107820. 10.1016/j.actatropica.2025.107820 40907780

[ref143] Notomi T, Okayama H, Masubuchi H, Yonekawa T, Watanabe K, Amino N and Hase T (2000) Loop-mediated isothermal amplification of DNA. *Nucleic Acids Research* 28(12), 1–7. 10.1093/nar/28.12.e63 10871386 PMC102748

[ref144] Noyes HA, Reyburn H, Bailey JW and Smith D (1998) A Nested-PCR-based schizodeme method for identifying Leishmania kinetoplast minicircle classes directly from clinical samples and its application to the study of the epidemiology of Leishmania tropica in Pakistan. *Journal of Clinical Microbiology* 36(10). 10.1128/jcm.36.10.2877-2881.1998 PMC105081 9738037

[ref145] Nzelu CO, Kato H and Peters NC (2019) Loop-mediated isothermal amplification (LAMP): an advanced molecular point-of-care technique for the detection of Leishmania infection. *PLoS Neglected Tropical Diseases* 13(11), e0007698. 10.1371/journal.pntd.0007698. 31697673 PMC6837287

[ref146] Otoo JA and Schlappi TS (2022) REASSURED multiplex diagnostics: a critical review and forecast. *Biosensors* 12, 124. 10.3390/bios12020124 35200384 PMC8869588

[ref147] Pan CY, Kijamnajsuk P and Chen JJ (2024) Portable loop-mediated isothermal amplification device with spectrometric detection for rapid pathogen identification. *Analytical Biochemistry* 694. 10.1016/j.ab.2024.115615 39002745

[ref148] Pang F, Zhang T, Dai F, Wang K, Jiao T, Zhang Z, Zhang L, Liu M, Hu P and Song J (2024) A handheld isothermal fluorescence detector for duplex visualization of aquatic pathogens via enhanced one-pot LAMP-PfAgo assay. *Biosensors and Bioelectronics* 254, 116187. 10.1016/j.bios.2024.116187 38518558

[ref149] Papadakis G, Pantazis AK, Fikas N, Chatziioannidou S, Tsiakalou V, Michaelidou K, Pogka V, Megariti M, Vardaki M, Giarentis K, Heaney J, Nastouli E, Karamitros T, Mentis A, Zafiropoulos A, Sourvinos G, Agelaki S and Gizeli E (2022) Portable real-time colorimetric LAMP-device for rapid quantitative detection of nucleic acids in crude samples. *Scientific Reports* 12(1), 3775. 10.1038/s41598-022-06632-7 35260588 PMC8904468

[ref150] Papaiakovou M, Waeschenbach A, Ajibola O, Ajjampur SS, Anderson RM, Bailey R, Benjamin-Chung J, Cambra-Pellejà M, Caro NR, Chaima D, Cimino RO, Cools P, Cossa A, Dunn J, Galagan S, Gandasegui J, Grau-Pujol B, Houlder EL, Ibikounlé M, Jenkins TP, Kalua K, Kjetland EF, Krolewiecki AJ, Levecke B, Luty AJ, MacDonald AS, Mandomando I, Manuel M, Martínez-Valladares M, Mejia R, Mekonnen Z, Messa A, Mpairwe H, Muchisse O, Muñoz J, Mwinzi P, Novela V, Odiere MR, Sacoor C, Walson JL, Williams SA, Witek-mcmanus S, Littlewood DTJ, Cantacessi C and Doyle SR (2025) Global diversity of soil-transmitted helminths reveals population-biased genetic variation that impacts diagnostic targets. *Nature Communications* 16(1), 6374. 10.1038/s41467-025-61687-0 PMC12246136 40640199

[ref151] Parajuli N and Prajapati B (2023) Use of mobile tele-dermatology in managing cutaneous leishmaniasis from a remote district of Nepal during the COVID 19 pandemic: A case series. *Tropical Doctor* 53(1), 158–160. 10.1177/00494755221136910 36344234 PMC9643109

[ref152] Patiño LH, Ballesteros N, Muñoz M, Jaimes J, Castillo-Castañeda AC, Madigan R, Paniz-Mondolfi A and Ramírez JD (2023) Validation of Oxford nanopore sequencing for improved New World Leishmania species identification via analysis of 70-kDA heat shock protein. *Parasites and Vectors* 16(1), 458. 10.1186/s13071-023-06073-9 38111024 PMC10726620

[ref153] Pereira LQ, Ferreira-Silva MM, Ratkevicius CMA, Gómez-Hérnandez C, De Vito FB, Tanaka SCSV, Rodrigues Júnior V and Moraes-Souza H (2021) Identification of Leishmania infantum in blood donors from endemic regions for visceral leishmaniasis. *Parasitology* 148(1), 110–114. 10.1017/S0031182020001936 33143775 PMC11010041

[ref154] Poirier AC, Riaño Moreno RD, Takaindisa L, Carpenter J, Mehat JW, Haddon A, Rohaim MA, Williams C, Burkhart P, Conlon C, Wilson M, McClumpha M, Stedman A, Cordoni G, Branavan M, Tharmakulasingam M, Chaudhry NS, Locker N, Fernando A, Balachandran W, Bullen M, Collins N, Rimer D, Horton DL, Munir M and La Ragione RM (2023) VIDIIA Hunter diagnostic platform: a low-cost, smartphone connected, artificial intelligence-assisted COVID-19 rapid diagnostics approved for medical use in the UK. *Frontiers in Molecular Biosciences* 10, 1144001. 10.3389/fmolb.2023.1144001 37842636 PMC10572354

[ref155] Pratlong F, Dereure J, Ravel C, Lami P, Balard Y, Serres G, Lanotte G, Rioux JA and Dedet JP (2009) Geographical distribution and epidemiological features of Old World cutaneous leishmaniasis foci, based on the isoenzyme analysis of 1048 strains. *Tropical Medicine and International Health* 14(9), 1071–1085. 10.1111/j.1365-3156.2009.02336.x 19624480

[ref156] Priya K, Kapoor A and Ramya M (2025) A paper-based loop-mediated isothermal amplification assay coupled with a portable heating device for point-of-care detection of syphilis in low-resource settings. *Analytical Methods* 17, 7420–30. 10.1039/D5AY00749F 40856541

[ref157] Puri M, Brar HK, Mittal N, Madan E, Srinivasan R, Rawat K, Moulik S, Chatterjee M, Gorthi SS, Muthuswami R and Madhubala R (2021) Rapid diagnosis of Leishmania infection with a portable loop-mediated isothermal amplification device. *Journal of Biosciences* 46(4), 92. 10.1007/s12038-021-00211-0 34635627 PMC8458559

[ref158] Quero FJ, Aidelberg G, Vielfaure H, de Kermadec YH, Cazaux S, Pandi A, Pascual-Garrigos A, Arce A, Sakyi S, Gaudenz U, Federici F, Molloy JC and Lindner AB (2025) qByte: An open-source isothermal fluorimeter for democratizing analysis of nucleic acids, proteins and cells. *PLOS Biology* 23(5), e3003199. 10.1371/journal.pbio.3003199 40435312 PMC12157786

[ref159] Rahimi BA, Ghatee MA, Habib MN, Farooqi K, Ritmeijer K, Hussain HS, Beg MA and Taylor WR (2025) Cutaneous leishmaniasis in Afghanistan. *Transactions of the Royal Society of Tropical Medicine and Hygiene* 77(29), 246. 10.1093/trstmh/traf028 PMC12342938 40331266

[ref160] Reimão JQ, Coser EM, Lee MR and Coelho AC (2020) Laboratory diagnosis of cutaneous and visceral leishmaniasis: current and future methods. *Microorganisms* 8(11), 1–30. 10.3390/microorganisms8111632 PMC7690623 33105784

[ref161] Reina AM, Mewa JC, Calzada JE and Saldaña A (2022) Characterization of Leishmania spp. causing cutaneous lesions with a negative parasitological diagnosis in Panama. *Tropical Medicine and Infectious Disease* 7(10), 282. 10.3390/tropicalmed7100282 36288023 PMC9609048

[ref162] Rihs JB, Vilela MT, Dos Santos JSC, Caldas S, Leite RS and Mol MPG (2025a) Exploring real-time PCR techniques for diagnosing leishmaniasis: Key insights from a systematic review. *Parasitology Research* 124(5), 54. 10.1007/s00436-025-08503-2 40397177 PMC12095430

[ref163] Rihs JB, Vilela MT, Dos Santos JSC, de Souza Filho JA, Caldas S, Leite RS and Mol MPG (2025b) February 1) qPCR as a tool for the diagnosis of visceral and cutaneous Leishmaniasis: a systematic review and meta-analysis. *Acta Parasitologica* 70(1), 16. 10.1007/s11686-024-00942-8. 39777570

[ref164] Rioboó-Legaspi P, González-López A, Beltrán-Sánchez JF, Cima-Cabal MD, García-Suárez MM, Sánchez AJG, Fernández-Otero T, Haro JG, Costa-Rama E and Fernández-Abedul MT (2024) Phenol red as electrochemical indicator for highly sensitive quantification of SARS-CoV-2 by loop-mediated isothermal amplification detection. *Talanta* 266, 124963. 10.1016/j.talanta.2023.124963 37517341

[ref165] Roberts T, Keddie SH, Rattanavong S, Gomez SR, Bradley J, Keogh RH, Bärenbold O, Falconer J, Mens PF, Hopkins H and Ashley EA (2023) Accuracy of the direct agglutination test for diagnosis of visceral leishmaniasis: A systematic review and meta-analysis. *BMC Infectious Diseases* 23(1), 782. 10.1186/s12879-023-08772-1 37946107 PMC10636880

[ref166] Rodriguez-Manzano J, Malpartida-Cardenas K, Moser N, Pennisi I, Cavuto M, Miglietta L, Moniri A, Penn R, Satta G, Randell P, Davies F, Bolt F, Barclay W, Holmes A and Georgiou P (2021) Handheld point-of-care system for rapid detection of SARS-CoV-2 extracted RNA in under 20 min. *ACS Central Science* 7(2), 307–317. 10.1021/acscentsci.0c01288 33649735 PMC7839415

[ref167] Roozbehani M, Tasbihi M, Keyvani H, Mosavizadeh L, Hasanpour H and Askari Z (2025) Exploring the potential of Taq Man quantitative PCR for the simulated diagnosis of cutaneous and visceral leishmaniasis in clinical samples in Iran. *Journal of Parasitic Diseases* 1-1. 10.1007/s12639-025-01806-2 PMC12602822 41230269

[ref168] Roy M, Ceruti A, Kobialka RM, Roy S, Sarkar D, Abd El Wahed A and Chatterjee M (2023) Evaluation of Recombinase Polymerase Amplification assay for monitoring parasite load in patients with kala-azar and post kala-azar dermal leishmaniasis. *PLoS Neglected Tropical Diseases* 17(4), e0011231. 10.1371/journal.pntd.0011231 37075066 PMC10115299

[ref169] Rune Stensvold C, Vang Høst A, Belkessa S and Vedel Nielsen H (2019) Evaluation of the NovaLisa^TM^ leishmania infantum IgG ELISA in a reference diagnostic laboratory in a non-endemic country. *Antibodies* 8(1), 20. 10.3390/antib8010020 31544826 PMC6640698

[ref170] Ruszova E, Vanek D, Stühmer W, Khaznadar Z and Subhashini N (2024) The utilization of the SaLux19-based loop-mediated isothermal amplification (LAMP) assay for the rapid and sensitive identification of minute amounts of a biological specimen. *Life* 14(5), 579. 10.3390/life14050579 38792600 PMC11122329

[ref171] Sadeghi A, Sadeghi M, Fakhar M, Zakariaei Z, Sadeghi M and Bastani R (2024) A deep learning-based model for detecting Leishmania amastigotes in microscopic slides: A new approach to telemedicine. *BMC Infectious Diseases* 24(1), 551. 10.1186/s12879-024-09428-4 38824500 PMC11144338

[ref172] Saengsawang N, Ruang-areerate P, Kaeothaisong N, Leelayoova S, Mungthin M, Juntanawiwat P, Hanyanunt P, Potisuwan P, Kesakomol P, Butsararattanagomen P, Wichaiwong P, Dungchai W and Ruang-areerate T (2023) Validation of quantitative loop-mediated isothermal amplification assay using a fluorescent distance-based paper device for detection of Escherichia coli in urine. *Scientific Reports* 13(1), 18781. 10.1038/s41598-023-46001-6 37907677 PMC10618465

[ref173] Salvador FGF, Oliveira LFA, Pimentel MIF, Lyra MR, Hasslocher-Moreno AM, Holanda MT, Varela MC, Silveira H and Valete CM (2025a) Telemedicine in the clinical care of Chagas disease and American cutaneous leishmaniasis: Pilot study in a public referral hospital in Brazil. *Frontiers in Public Health* 13, 1616368. 10.3389/fpubh.2025.1616368 40642251 PMC12243272

[ref174] Salvador FGF, Wakimoto MD, Duarte CCJ, Lapão LV, Silveira H and Valete CM (2025b) Telemedicine in the Clinical Care of Neglected Tropical Diseases: A Scoping Review. *PLOS Neglected Tropical Diseases* 19, e0012431. 10.1371/journal.pntd.0012431 40198712 PMC12011287

[ref175] Sanmoung W, Sawangjaroen N, Jitueakul S, Buncherd H, Tun AW, Thanapongpichat S and Imwong M (2023) Application of loop-mediated isothermal amplification combined with lateral flow assay visualization of Plasmodium falciparum kelch 13 C580Y mutation for artemisinin resistance detection in clinical samples. *Acta Tropica* 246, 106998. 10.1016/j.actatropica.2023.106998 37544396 PMC10465885

[ref176] Schwenkenbecher JM, Wirth T, Schnur LF, Jaffe CL, Schallig H, Al-Jawabreh A, Hamarsheh O, Azmi K, Pratlong F and Schönian G (2006) Microsatellite analysis reveals genetic structure of Leishmania tropica. *International Journal for Parasitology* 36(2), 237–246. 10.1016/j.ijpara.2005.09.010 16307745

[ref177] Shah KG, Roller M, Kumar S, Bennett S, Heiniger E, Looney K, Buser J, Bishop JD and Yager P (2023) Disposable platform for bacterial lysis and nucleic acid amplification based on a single USB-powered printed circuit board. *PLoS ONE* 18(4), e0284424. 10.1371/journal.pone.0284424 37099532 PMC10132542

[ref178] Sharma S, Caputi M and Asghar W (2024) Development of a diagnostic microfluidic chip for SARS-CoV-2 detection in saliva and nasopharyngeal samples. *Viruses* 16(8), 1190. 10.3390/v16081190 39205164 PMC11360425

[ref179] Sherrill-Mix S, Hwang Y, Roche AM, Glascock A, Weiss SR, Li Y, Haddad L, Deraska P, Monahan C, Kromer A, Graham-Wooten J, Taylor LJ, Abella BS, Ganguly A, Collman RG, Van Duyne GD and Bushman FD (2021) Detection of SARS-CoV-2 RNA using RT-LAMP and molecular beacons. *Genome Biology* 22(1), 169. 10.1186/s13059-021-02387-y 34082799 PMC8173101

[ref180] Shi L, Pang Z, Yu J, Zhu J, Xie X, Xie S, Gu L, Hu W, Xu H, Li L, Tao J and Wang M (2025) Development of a portable multi-step microfluidic device for point-of-care nucleic acid diagnostics. *Analytica Chimica Acta* 1336, 343518. 10.1016/j.aca.2024.343518 39788671

[ref181] Shrestha A, Mishra A, Mishra A, Shrestha R and Shrestha R (2024) Uncommon presentation of cutaneous leishmaniasis: Late-onset facial involvement after a decade - a rare case report. *Oxford Medical Case Reports* 2024(1), omad141. 10.1093/omcr/omad141 38292157 PMC10823326

[ref182] Sikorska K, Gesing M, Olszański R, Roszko-Wysokińska A, Szostakowska B and Van Damme-Ostapowicz K (2022) Misdiagnosis and inappropriate treatment of cutaneous leishmaniasis: A case report. *Tropical Diseases, Travel Medicine and Vaccines* 8(1), 18. 10.1186/s40794-022-00175-5 35909173 PMC9341103

[ref183] Silgado A, Armas M, Sánchez-Montalvá A, Goterris L, Ubals M, Temprana-Salvador J, Aparicio G, Chicharro C, Serre-Delcor N, Ferrer B, Molina I, García-Patos V, Pumarola T and Sulleiro E (2021) Changes in the microbiological diagnosis and epidemiology of cutaneous leishmaniasis in real-time pcr era: A six-year experience in a referral center in barcelona. *PLoS Neglected Tropical Diseases* 15(11), e0009884. 10.1371/journal.pntd.0009884 34758023 PMC8580242

[ref184] Soares ARC, de Faria VCS and de Avelar DM (2024) Development and accuracy evaluation of a new loop-mediated isothermal amplification assay targeting the HSP70 gene for the diagnosis of cutaneous leishmaniasis. *PLoS ONE* 19(8), e0306967. 10.1371/journal.pone.0306967 39172895 PMC11340985

[ref185] Sohn H, Puri L, Nguyen NAT, Van’t Hoog AH, Nguyen VAT, Nliwasa M and Nabeta P (2019) Cost and affordability analysis of TB-LAMP and Xpert MTB/RIF assays as routine diagnostic tests in peripheral laboratories in Malawi and Vietnam. *Journal of Global Health Science* 1(1), e22. 10.35500/jghs.2019.1.e22

[ref186] Solomon M, Greenberger S, Milner M, Pavlotzky F, Barzilai A, Schwartz E, Hadayer N and Baum S (2022) Efficacy of systemic treatment for leishmania tropica cutaneous Leishmaniasis. *Acta Dermato-Venereologica* 102, 2079. 10.2340/actadv.v102.2079 35229163 PMC9574677

[ref187] Song M, Hong SG and Lee LP (2023) Multiplexed ultrasensitive sample-to-answer RT-LAMP chip for the identification of SARS-CoV-2 and influenza viruses. *Advanced Materials* 35(10), 2207138. 10.1002/adma.202207138 36398425

[ref188] Spurlock N, Alfaro R, Gabella WE, Chugh K, Pask ME, Baudenbacher F and Haselton FR (2025) Achievement of 15-Minute Adaptive PCR Benchmark with 1370 nm Laser Heating. *Biosensors* 15(4), 258. 10.3390/bios15040258 40277572 PMC12026111

[ref189] Steyve N, Steve P, Ghislain M, Ndjakomo S and Pierre E (2022) Optimized real-time diagnosis of neglected tropical diseases by automatic recognition of skin lesions. *Informatics in Medicine Unlocked* 33, 101078. 10.1016/j.imu.2022.101078

[ref190] Taslimi Y, Habibzadeh S, Goyonlo VM, Akbarzadeh A, Azarpour Z, Gharibzadeh S, Shokouhy M, Persson J, Harandi AM, Mizbani A and Rafati S (2023) Tape-disc-loop-mediated isothermal amplification (TD-LAMP) method as noninvasive approach for diagnosis of cutaneous leishmaniasis caused by L. tropica. *Heliyon* 9(11), e21397. 10.1016/j.heliyon.2023.e21397 38027876 PMC10643283

[ref191] Taye B, Gebrie H, Bogale A, Getu E and Churiso G (2025) A novel pan-Leishmania loop-mediated isothermal amplification (Loopamp) assay for diagnosis of cutaneous and visceral leishmaniasis: A systematic review and meta-analysis. *BMC Infectious Diseases* 25(1), 718. 10.1186/s12879-025-11091-2 40383753 PMC12087146

[ref192] Taye B, Melkamu R, Tajebe F, Ibarra-Meneses AV, Adane D, Atnafu S, Adem M, Adane G, Kassa M, Asres MS, van Griensven J, van Henten S and Pareyn M (2024) Evaluation of Loopamp Leishmania detection kit for the diagnosis of cutaneous leishmaniasis in Ethiopia. *Parasites and Vectors* 17(1), 431. 10.1186/s13071-024-06475-3 39407317 PMC11481786

[ref193] Tekle E, Dese K, Girma S, Adissu W, Krishnamoorthy J and Kwa T (2024) DeepLeish: A deep learning based support system for the detection of Leishmaniasis parasite from Giemsa-stained microscope images. *BMC Medical Imaging* 24(1), 152. 10.1186/s12880-024-01333-1 38890604 PMC11186139

[ref194] Tesfaye M, Gebressilassie A, Mekonnen Z, Zeynudin A and Yewhalaw D (2025) Molecular epidemiology, distribution, and determinants of cutaneous leishmaniasis in Northeast Ethiopia. *Scientific Reports* 15(1), 25682. 10.1038/s41598-025-02107-7 40664709 PMC12264162

[ref195] Thakur S, Joshi J and Kaur S (2020) Leishmaniasis diagnosis: An update on the use of parasitological, immunological and molecular methods. *Journal of Parasitic Diseases* 44(2), 253–272. 10.1007/s12639-020-01212-w 32419743 PMC7223249

[ref196] Thorapalli Muralidharan S, Hanze M, Ainla A, Möller B, Hamedi MM and Toldrà A (2025) Lab-on-PCB with integrated DNA amplification and electroanalytical detection for point-of-care diagnostics. *Scientific Reports* 15(1), 32418. 10.1038/s41598-025-12364-1 40940334 PMC12432110

[ref197] Tomlinson JA, Dickinson MJ and Boonham N (2010) Detection of Botrytis cinerea by loop-mediated isothermal amplification. *Letters in Applied Microbiology* 51(6), 650–657. 10.1111/j.1472-765X.2010.02949.x 21029140

[ref198] Torrico MC, Fernández-Arévalo A, Ballart C, Solano M, Rojas E, Ariza E, Tebar S, Lozano D, Abras A, Gascón J, Picado A, Muñoz C, Torrico F and Gállego M (2022) Tegumentary leishmaniasis by Leishmania braziliensis complex in Cochabamba, Bolivia including the presence of L. braziliensis outlier: Tegumentary leishmaniasis in Cochabamba, Bolivia. *Transboundary and Emerging Diseases* 69(4), 2242–2255. 10.1111/tbed.14228 34232559

[ref199] Touria HS, Kheira S, Nori M, Assia B, Amel L and Fadi B (2018) Epidemiology of infantile visceral Leishmaniasis in Western Algerian and the convenience of serum for the disease diagnosis by PCR and Immunochromatography. *International Journal of Molecular and Cellular Medicine* 7(1), 32–43. 10.22088/IJMCM.BUMS.7.1.32 30234071 PMC6134421

[ref200] Trinh KTL, Park SY, Lee H and Lee NY (2025) A LEGO®-inspired pipette-free approach for a fully-integrated molecular diagnostic kit isothermally operated at near body temperature for the detection of antibacterial resistance. *The Analyst* 150(19), 4274–4284. 10.1039/D5AN00628G 40888462

[ref201] Ullah W, Khan A, Niaz S, Al-Garadi MA, Nasreen N, Swelum AA and Said MB (2024) Epidemiological survey, molecular profiling and phylogenetic analysis of cutaneous leishmaniasis in Khyber Pakhtunkhwa, Pakistan. *Transactions of the Royal Society of Tropical Medicine and Hygiene* 118(4), 273–286. 10.1093/trstmh/trad086 38055843

[ref202] Van den Broeck F, Heeren S, Maes I, Sanders M, Cotton JA, Cupolillo E, Alvarez E, Garcia L, Tasia M, Marneffe A, Dujardin JC and Van der AG (2023) Genome Analysis of Triploid Hybrid Leishmania Parasite from the Neotropics. *Emerging Infectious Diseases* 29(5), 1076–1078. 10.3201/eid2905.221456 37081624 PMC10124652

[ref203] van Dijk NJ, Hagos DG, Huggins DM, Carrillo E, Ajala S, Chicharro C, Kiptanui D, Solana JC, Abner E, Wolday D and Schallig HDFH (2024) Simplified molecular diagnosis of visceral leishmaniasis: laboratory evaluation of miniature direct-on-blood PCR nucleic acid lateral flow immunoassay. *PLoS Neglected Tropical Diseases* 18(5), e0011637. 10.1371/journal.pntd.0011637 38713648 PMC11075898

[ref204] van Henten S, Diro E, Tesfaye AB, Tilahun Zewdu F, van Griensven J and Enbiale W (2025) Ambiguities in cutaneous leishmaniasis classification and the need for consensus: experience from Ethiopia. *PLOS Neglected Tropical Diseases* 19(8), e0013458. 10.1371/journal.pntd.0013458 40845055 PMC12396759

[ref205] Van Henten S, Fikre H, Melkamu R, Dessie D, Mekonnen T, Kassa M, Bogale T, Mohammed R, Cnops L, Vogt F, Pareyn M and Van Griensven J (2022) Evaluation of the CL detect rapid test in Ethiopian patients suspected for Cutaneous Leishmaniasis. *PLoS Neglected Tropical Diseases* 16(1), e0010143. 10.1371/JOURNAL.PNTD.0010143 35041672 PMC8797207

[ref206] van Henten S, Kassa M, Fikre H, Melkamu R, Mekonnen T, Dessie D, Mulaw T, Bogale T, Engidaw A, Yeshanew A, Cnops L, Vogt F, Moons KGM, van Griensven J and Pareyn M (2024) Evaluation of less invasive sampling tools for the diagnosis of cutaneous Leishmaniasis. *Open Forum Infectious Diseases* 11(4), ofae113. 10.1093/ofid/ofae113 38560600 PMC10977625

[ref207] Verma S, Avishek K, Sharma V, Negi NS, Ramesh V and Salotra P (2013) Application of loop-mediated isothermal amplification assay for the sensitive and rapid diagnosis of visceral leishmaniasis and post-kala-azar dermal leishmaniasis. *Diagnostic Microbiology and Infectious Disease* 75(4), 390–395. 10.1016/j.diagmicrobio.2013.01.011 23433714

[ref208] Vink MMT, Nahzat SM, Rahimi H, Buhler C, Ahmadi BA, Nader M, Zazai FR, Yousufzai AS, van Loenen M, Schallig HDFH, Picado A and Cruz I (2018) Evaluation of point-of-care tests for cutaneous leishmaniasis diagnosis in Kabul, Afghanistan. *EBioMedicine* 37, 453–460. 10.1016/j.ebiom.2018.10.063 30396855 PMC6286266

[ref209] Wan L, Chen T, Gao J, Dong C, Wong AHH, Jia Y, Mak PI, Deng CX and Martins RP (2017) A digital microfluidic system for loop-mediated isothermal amplification and sequence specific pathogen detection. *Scientific Reports* 7(1), 14586. 10.1038/s41598-017-14698-x 29109452 PMC5673945

[ref210] Wang F, Hu F, Zhang Y, Li X, Ma Q, Wang X and Peng N (2024) A novel high-throughput sample-in-result-out device for the rapid detection of viral nucleic acids. *Biosensors* 14(11), 549. 10.3390/bios14110549 39590008 PMC11591587

[ref211] Wiggins TJ, Peng R, Bushnell RV, Tobin JK, MacLeod DA, Du K, Tobin GJ and Dollery SJ (2024) Instrument-free point-of-care diagnostic for Leishmania Parasites. *Diagnostics* 14(23), 2744. 10.3390/diagnostics14232744 39682651 PMC11640444

[ref212] Ntuli Malecela Mwelecele, World Health Organisation (2021) Ending the neglect to attain the Sustainable Development Goals: A road map for neglected tropical diseases 2021–2030. Geneva, Switzerland: World Health Organization. https://www.who.int/publications/i/item/9789240010352 (accessed 19 August 2025).

[ref213] World Health Organisation (2022) *Module 2: Screening WHO Operational Handbook on Tuberculosis Systematic Screening for Tuberculosis Disease*. Geneva, Switzerland: World Health Organization. 124, (accessed 19 August 2025).

[ref214] Wormald BW, Moser N, deSouza NM, Mantikas KT, Malpartida-Cardenas K, Pennisi I, Ind TEJ, Vroobel K, Kalofonou M, Rodriguez-Manzano J and Georgiou P (2022) Lab-on-chip assay of tumour markers and human papilloma virus for cervical cancer detection at the point-of-care. *Scientific Reports* 12(1), 8750. 10.1038/s41598-022-12557-y 35610285 PMC9128326

[ref215] Wu W, Biyani M, Hirose D and Takamura Y (2023) Rapid and highly sensitive detection of leishmania by combining recombinase polymerase amplification and solution-processed oxide thin-film transistor technology. *Biosensors* 13(8), 765. 10.3390/bios13080765 37622851 PMC10452724

[ref216] Xiao B, Zhou T, Wang N, Zhang J, Sun X, Chen J, Huang F, Wang J, Li N and Chen A (2024) Toothpick DNA extraction combined with handheld LAMP microfluidic platform for simple and rapid meat authentication. *Food Chemistry* 460, 140659. 10.1016/j.foodchem.2024.140659 39111039

[ref217] Xue H, Cao M, Wang S, Fei Y, Xiong X and Yang Y (2024) Visual and rapid detection of escolar (Lepidocybium flavobrunneum) using loop mediated isothermal amplification in conjunction with a specific molecular beacon probe. *Food Chemistry* 432, 137262. 10.1016/j.foodchem.2023.137262 37643514

[ref218] Yang K, Bi M and Mo X (2024) CRISPR/Cas12a, combined with recombinase polymerase amplification (RPA) reaction for visual detection of Leishmania species. *Microchemical Journal* 207, 112283. 10.1016/j.microc.2024.112283

[ref219] Yang T, Xue L, Luo Z, Lin J, Zhang X, Xiao F, Liu Y, Li D and Lin X (2025a) Sensitivity-enhanced hydrogel digital RT-LAMP with in situ enrichment and interfacial reaction for norovirus quantification in food and water. *Journal of Hazardous Materials* 488, 137325. 10.1016/j.jhazmat.2025.137325 39864200

[ref220] Yang T, Zhang X, Yan Y, Liu Y, Lin X and Li W (2025b) Artificial intelligence-driven quantification of antibiotic-resistant Bacteria in food by color-encoded multiplex hydrogel digital LAMP. *Food Chemistry* 468, 142304. 10.1016/j.foodchem.2024.142304 39667227

[ref221] Yizengaw E, Takele Y, Franssen S, Gashaw B, Yimer M, Adem E, Nibret E, Yismaw G, Cruz Cervera E, Ejigu K, Tamiru D, Munshea A, Müller I, Weller R, Cotton JA and Kropf P (2024) Investigation of parasite genetic variation and systemic immune responses in patients presenting with different clinical presentations of cutaneous leishmaniasis caused by Leishmania aethiopica. *Infectious Diseases of Poverty* 13(1), 76. 10.1186/s40249-024-01244-x 39415297 PMC11484111

[ref222] Yotsu RR, Ding Z, Hamm J and Blanton RE (2023) Deep learning for AI-based diagnosis of skin-related neglected tropical diseases: A pilot study. *PLoS Neglected Tropical Diseases* 17(8), e0011230. 10.1371/journal.pntd.0011230 37578966 PMC10449179

[ref223] Yu L-S, Rodriguez-Manzano J, Moser N, Moniri A, Malpartida-Cardenas K, Miscourides N, Sewell T, Kochina T, Brackin A, Rhodes J, Holmes AH, Fisher MC and Georgiou P (2020) Rapid detection of azole-resistant aspergillus fumigatus in clinical and environmental isolates by use of a lab-on-a-chip diagnostic system. *Journal of Clinical Microbiology* 58(11), 10-128. 10.1128/JCM.00843-20 PMC7587087 32907990

[ref224] Zare M, Akbarialiabad H, Parsaei H, Asgari Q, Alinejad A, Bahreini MS, Hosseini SH, Ghofrani-Jahromi M, Shahriarirad R, Amirmoezzi Y, Shahriarirad S, Zeighami A and Abdollahifard G (2022) A machine learning-based system for detecting leishmaniasis in microscopic images. *BMC Infectious Diseases* 22(1), 48. 10.1186/s12879-022-07029-7 35022031 PMC8754077

[ref225] Zeinali M, Mohebali M, Shirzadi MR, Hassanpour G, Behkar A, Gouya MM, Samiee SM and Malekafzali H (2023) Integration and evaluation of cutaneous leishmaniasis laboratory diagnosis in the primary. *Eastern Mediterranean Health Journal* 29(10), 810–818. 10.26719/emhj.23.105 37947232

[ref226] Zeng M, Wang X, Tan Z, Guo W, Deng Y, Li S, Nie L, He N and Chen Z (2025a) A novel rapid detection method for mycobacterium tuberculosis based on scattering-light turbidity using loop-mediated isothermal amplification. *Biosensors* 15(3), 162. 10.3390/bios15030162 40136959 PMC11939914

[ref227] Zeng W, Li B, Chen Y, Lin B, Gu X, Liu P and Zhang Y (2025b) A finger-actuated microfluidic system for point-of-care detection of SARS-CoV-2 and Influenza A. *Analytical Chemistry* 97(34), 18469–18478. 10.1021/acs.analchem.5c01644 40825145

[ref228] Zewdu FT, Tessema AM, Zerga AA, van Henten S and Lambert SM (2022) Effectiveness of intralesional sodium stibogluconate for the treatment of localized cutaneous leishmaniasis at Boru Meda general hospital, Amhara, Ethiopia: pragmatic trial. *PLoS Neglected Tropical Diseases* 16(9), e0010578. 10.1371/JOURNAL.PNTD.0010578 36084153 PMC9491591

[ref229] Zhang J, Li T, Zhou P, Zhu L, Lin X and Su B (2025a) Hydrogel-based electrochemical LAMP sensor for rapid, contamination-free and ultrasensitive point-of-care detection of Vibrio parahaemolyticus. *Chemical Communications* 61(42), 7660–7663. 10.1039/d5cc01526j 40302700

[ref230] Zhang J, Xu L, Sheng Z, Zheng J, Chen W, Hu Q and Shen F (2024) Combination-lock SlipChip integrating nucleic acid sample preparation and isothermal LAMP Amplification for the detection of SARS-CoV-2. *ACS Sensors* 9(2), 646–653. 10.1021/acssensors.3c01727 38181090

[ref231] Zhang R, Dai D, Cao Y, Huang Y, Wang K, Zou G, Liu C, Luo Z and Yi C (2025b) A portable smartphone-based electrochemiluminescence device integrated with bipolar electrode detection chip for multi-target respiratory pathogen detection. *Biosensors and Bioelectronics* 288, 117783. 10.1016/j.bios.2025.117783 40660655

[ref232] Zhang SX, Yang GB, Sun JY, Li YJ, Yang J, Wang JC and Deng Y (2025c) Global, regional, and national burden of Visceral leishmaniasis, 1990–2021: Findings from the global burden of disease study 2021. *Parasites and Vectors* 18(1), 157. 10.1186/s13071-025-06796-x 40287729 PMC12032768

[ref233] Zhao S, Zhang Y, Wang Y, Ren Z, Wei P, Zhang T, Peng R, Zhou H and Hu F (2025) Sample-to-answer nucleic acid detection using a fully integrated microdevice for nucleic acid extraction and smartphone-based droplet digital RPA/CRISPR. *Biosensors and Bioelectronics* 289, 117886. 10.1016/j.bios.2025.117886 40840128

[ref234] Zou Y, Mason MG and Botella JR (2023) A low-cost, portable, dual-function readout device for amplification-based point-of-need diagnostics. *Applied and Environmental Microbiology* 89(12), e00902–23. 10.1128/aem.00902-23 38047632 PMC10734478

