# Supplementary material for: Molecular diagnostics for cutaneous leishmaniasis: progress towards fulfilling the WHO target product profile
Source: Parasitology. 2025 Dec 26;153(3):333–51. doi: 10.1017/S0031182025101467 (PMC13215747; doi:10.1017/S0031182025101467)
Supplement: Jarzabek and Denny supplementary material [file S0031182025101467sup001.docx]

## Supplementary Information

**Table S1.** New isothermal amplification devices published in the years 2021-2025.

| **Device Name** | **Detection** | **Real-Time/**  **End-Point** | **Quantitative** | **Technique** | **Sample Tested** | **Format** | **Capacity** | **Cost** | **Reference** |
| --- | --- | --- | --- | --- | --- | --- | --- | --- | --- |
| VH6 | Colorimetric | Real-Time | Yes | LAMP | Covid-19 | PCR Strip | 8 | 2000 GBP | (Poirier *et al.* 2023) |
| Pebble | Colorimetric | Real-Time | Yes | LAMP | Covid-19, West Nile Virus | PCR Strip | 8 | 750 EUR | (Botha *et al.* 2024; Khedhiri *et al.* 2024; Papadakis *et al.* 2022) |
| Dragonfly | Colorimetric | End-Point | No | LAMP | MPXV, OPXV, VZV, HSV | PCR Strip | 32 | 100 GBP | (Cavuto *et al.* 2025) |
| n/a | Colorimetric | End-Point | Yes | LAMP | Covid-19 | PCR Strip | 8 | 100 USD | (Camargo *et al.* 2024) |
| SlipChip | Colorimetric | End-Point | Yes | LAMP | Covid-19, Influenza, M. pneumonia, HRV | PCR Tube | 2 | 10 USD | (Zhang *et al.* 2024) |
| n/a | Colorimetric | Real-Time | Yes | LAMP | *C. sativa* | Microfluidic | 1 | n/a | (Miyajima *et al.* 2025) |
| n/a | Colorimetric | Real-Time | Yes | LAMP | MPXV | Microfluidic | 1 | n/a | (Birtek *et al.* 2025) |
| n/a | Colorimetric | Real-Time | Yes | LAMP | Covid-19, Influenza | Microfluidic | 1 | 127 USD | (Zeng *et al.* 2025b) |
| n/a | Colorimetric | Real-Time | Yes | LAMP | *S. aureus* | Microfluidic | 1 | n/a | (Lee *et al.* 2025) |
| n/a | Colorimetric | Real-Time | Yes | LAMP | *Z. mays* (GMO), *G. max* (GMO) | PAD | 26 | 1500 USD | (Ahmed *et al.* 2025) |
| n/a | Colorimetric | End-Point | No | LAMP | *E. faecium, A. baumannii* | PAD | 2 | n/a | (Trinh *et al.* 2025) |
| n/a | Colorimetric | End-Point | No | LAMP | *T. pallidum* | PAD | 3 | n/a | (Priya *et al.* 2025) |
| n/a | Colorimetric | End-Point | No | RPA | HPV | PAD | 1 | 1000USD | (Kundrod *et al.* 2023) |
| SaLux19 | Fluorescent | Real-Time | Yes | LAMP | *S. scrofa* | PCR Tube | 4 | n/a | (Ruszova *et al.* 2024) |
| WeD-1 | Fluorescent | Real-Time  End-Point | Yes | LAMP | LMBV, EHP | PCR Strip | 8 | 500 USD | (Pang *et al.* 2024) |
| qByte | Fluorescent | Real-Time | Yes | LAMP | *S. mansoni*, *S. typhi*, Zika Virus | PCR Strip | 8 | 60 USD | (Quero *et al.* 2025) |
| WeD-mini | Fluorescent | Real-Time  End-Point | Yes | LAMP | Covid-19, Influenza, *C. felis*, *M. felis*,  *B. bronchiseptica*, FCV, FHV | PCR Tube | 2 | n/a | (Dai *et al.* 2025) |
| n/a | Fluorescent | End-Point | No | LAMP | *L. donovani* | PCR Tube | 9 | n/a | (Puri *et al.* 2021) |
| n/a | Fluorescent | Real-Time | Yes | LAMP | *S. typhimurium*, PLRV, TSWV,  *P. syringae* | PCR Tube | 1 | <1000 USD | (Pan *et al.* 2024) |
| White Pearl | Fluorescent | End-Point | Yes | LAMP | *E. coli*, *K. pneumoniae*, *P. aeruginosa*, *A. baumannii* | PCR Strip | 8 | 210 USD | (Castellanos *et al.* 2024) |
| n/a | Fluorescent | Real-Time | Yes | RPA | Covid-19, African Swine Fever Virus | PCR Tube | 6 | 1923 USD | (Wang *et al.* 2024) |
| n/a | Fluorescent | Real-Time | Yes | RPA | Covid-19, Influenza | PCR Tube | 4 | n/a | (Hu *et al.* 2025) |
| iNAAT | Fluorescent | Real-Time | Yes | LAMP | Covid-19, Influenza, RSV | PCR Strip | 8 | n/a | (Kshirsagar *et al.* 2025) |
| n/a | Fluorescent | Real-Time | Yes | LAMP | Covid-19 | PCR Tube | 9 | n/a | Cvodi(Lin et al. 2025b) |
| Dr Diagnose | Fluorescent  Turbidity | End-Point | No | LAMP | *F. oxysporum* | PCR Tube | 1 | 5 USD | (Zou *et al.* 2023) |
| n/a | Fluorescent | Real-Time | Yes | LAMP | *S. scrofa* | Microfluidic | 8 | 865.75 USD | (Xiao *et al.* 2024) |
| n/a | Fluorescent | Real-Time | Yes | LAMP | Covid-19 | Microfluidic | 3 | n/a | (Kumar *et al.* 2025) |
| n/a | Fluorescent | Real-Time | Yes | LAMP | Covid-19, *T. vaginalis* | Microfluidic | 1 | n/a | (Shi *et al.* 2025) |
| n/a | Fluorescent | End-Point | Yes | LAMP | *E. coli*, *K. pneumoniae*, *P. aeruginosa*, *A. baumannii* | Microfluidic | 1 | n/a | (Cui *et al.* 2025b) |
| CARE | Fluorescent | Real-Time | Yes | RPA | EHP, IHHNV, AHND, SHIV, CMNV, HPPV, WSSV, AIV, NDV, IBV, ILTV, FadV, FPV, FMDV, ASFV, PRRSV, PRV, JEV, PCV, PEDV | Microfluidic | 8 | n/a | (Ge *et al.* 2025) |
| n/a | Fluorescent | Real-Time | Yes | RPA | *C. trachomatis*, *T. pallidum*, *U. urealyticum*, HSV | Microfluidic | 6 | n/a | (Lin *et al.* 2025a) |
| n/a | Fluorescent | Real-Time | Yes | LAMP | EBOV, MARV, RVFV, LASV, YFV, MPXV, VARV | Microfluidic | 8 | n/a | (Li *et al.* 2025) |
| n/a | Fluorescent | End-Point | Yes | RAA | *E. coli*, *S. typhimurium*, *L. monocytogenes* | Microfluidic | 1 | n/a | (Jin *et al.* 2025) |
| UbiNAAT | Fluorescent | Real-Time | Yes | LAMP | *C. trachomatis*, *N. gonorrhoeae* | Microfluidic | 2 | 3 USD | (Heiniger *et al.* 2025) |
| n/a | Fluorescent | Real-Time | Yes | RPA | Covid-19 | Microfluidic | 1 | n/a | (Zhao *et al.* 2025) |
| n/a | Fluorescent | Real-Time | Yes | SDA | *S. aureus* | Microfluidic | 1 | 11 USD | (Shah *et al.* 2023) |
| MUSAL | Fluorescent | Real-Time | Yes | LAMP | Covid-19, Influenza | Microfluidic | 6 | n/a | (Song *et al.* 2023) |
| n/a | Fluorescent | Real-Time | Yes | LAMP | *C. difficile* | Microfluidic | 1 | n/a | (Bachmann *et al.* 2024) |
| FINDR | Fluorescent | End-Point | Yes | LAMP | *M. tuberculosis* | PAD | 1 | n/a | (Dong *et al.* 2024b) |
| n/a | Fluorescent | End-Point | Yes | LAMP | *E. coli* | PAD | 1 | n/a | (Saengsawang *et al.* 2023) |
| n/a | Fluorescent | Real-Time | Yes | LAMP | Covid-19 | PAD | 6 | 160 USD | (Dong *et al.* 2023) |
| n/a | Fluorescent | Real-Time | Yes | RPA | HPV | PAD | 1 | <1 USD | (Sharma *et al.* 2024) |
| n/a | Fluorescent | End-Point | Yes | LAMP | Covid-19 | PAD | 2 | n/a | (Ho *et al.* 2023) |
| n/a | Fluorescent | End-Point | Yes | LAMP | Human norovirus | Hydrogel | 6 | n/a | (Yang *et al.* 2025a) |
| n/a | Fluorescent | Real-Time | Yes | LAMP | *S. aureus*, *E. coli* | Hydrogel | 3 | n/a | (Yang *et al.* 2025b) |
| n/a | Fluorescent | Real-Time | Yes | LAMP | *H. pylori* | Hydrogel | 1 | n/a | (Cui *et al.* 2025a) |
| n/a | Electrochemical | End-Point | Yes | LAMP | *P. vivax* | PCR Tube | 1 | n/a | (Dong *et al.* 2024a) |
| n/a | Colorimetric  Electrochemical | End-Point | Yes | LAMP | Covid-19 | PCR Tube | 1 | n/a | (Rioboó-Legaspi *et al.* 2024) |
| E-LAMP | Colorimetric Electrochemical | End-Point | Yes | LAMP | *S. aureus* | PCR Tube | 1 | n/a | (de Lima *et al.* 2025) |
| n/a | Electrochemical | End-Point | Yes | RPA | *L. braziliensis* | Microfluidic | 1 | n/a | (Wu *et al.* 2023) |
| n/a | Electrochemical | Real-Time | Yes | LAMP | *V. parahaemolyticus* | Hydrogel | 1 | n/a | (Zhang *et al.* 2025a) |
| n/a | Electrochemical | End-point | Yes | LAMP | Covid-19 | PCB | 1 | <10 USD | (Thorapalli Muralidharan *et al.* 2025) |
| n/a | Electrochemical | End-Point | Yes | LAMP | Influenza, HadV | Chip | 4 | n/a | (Zhang *et al.* 2025b) |
| n/a | Electrochemical | Real-Time | Yes | CHA | Covid-19 | Chip | 1 | n/a | (Kim *et al.* 2024) |
| Lacewing | Electrochemical | Real-Time | Yes | LAMP | *P. falciparum, P. ovale, P. malariae, P. vivax, P. cynomolgi, P. knowlesi, A. fumigatus,* Covid-19, DENV, HPV | Chip | 1 | n/a | (Malpartida-Cardenas *et al.* 2023; Moser *et al.* 2022; Rodriguez-Manzano *et al.* 2021; Wormald *et al.* 2022; Yu *et al.* 2020) |
| n/a | Electrochemical | Real-Time | Yes | RCA | *E. coli, P. aeruginosa, S. paucimobilis, S. capitis, N. brasiliensis, S. dysgalactiae, A. jandaei, A. baumannii, B. subtilis, S. anginosus, R. ornithinolytica, S. pyogenes, C. werkmanii* | Chip | 1 | n/a | (Liao *et al.* 2024) |
| n/a | Colorimetric Electrochemical | End-Point | Yes | LAMP | Covid-19 | Chip | 5 | n/a | (Kim *et al.* 2024) |
| StratoLAMP | Turbidity | Real-Time | Yes | LAMP | Human Papillomavirus | Droplet Digital | 2 | n/a | (Jin *et al.* 2024) |
| n/a | Turbidity | Real-Time | Yes | LAMP | *M. tuberculosis* | PCR Tube | 1 | n/a | (Zeng *et al.* 2025a) |
| n/a | Raman  Scattering | End-Point | Yes | LAMP | *S. aureus*, *P. aeruginosa*, *E. coli* | PCR Tube | 3 | n/a | (Jiang *et al.* 2025) |

Avian Influenza virus; AHND - Acute hepatopancreatic necrosis disease; CHA – catalytic hairpin assembly; CMNV - Covert mortality nodavirus; DENV – Dengue virus; EBOV – Ebola virus; EHP - Enterocytozoon hepatopenaei; ; FadV - Fowl adenovirus; FCV - Feline calicivirus; FHV - Feline herpesvirus; FMDV - Foot and Mouth Disease virus; FPV - Fowlpox virus; IHHNV - Infectious hypodermal hematopoietic necrosis virus; ILTV - Infectious laryngotracheitis virus; HadV – Human adenovirus; HPPV - Hepatopancreatic parvovirus; HRV – Human rhinovirus; HSV - Herpes Simplex virus; IBV - Infectious bronchitis virus; JEV - Japanese encephalitis virus; LAMP – loop-mediated isothermal amplification; LASV – Lassa virus; LMBV -Largemouth bass virus; MARV – Marburg virus; MPXV - Monkeypox virus; NDV - Newcastle Disease virus; OPXV - Orthopox virus; PCV - Porcine circovirus; PEDV - Porcine epidemic diarrhea virus; PLRV - Potato leafroll virus; PRRSV - Porcine reproductive and respiratory syndrome virus; PRV - Pseudorabies virus; RAA – recombinase-aided amplification; RCA – rolling circle amplification; RPA – recombinase polymerase amplification; RSV – respiratory syncytial virus; RVFV – Rift Valley fever virus; SDA – strand displacement amplification; SHIV - Shrimp hemocyte iridescent virus; TSWV - Tomatto spotted wilt virus; VZV - Varicella-Zoster virus; VARV – variola virus; WSSV - White Spot Syndrome virus; YFV – yellow fever virus
